# Genome of Paulownia (*Paulownia fortunei*) illuminates the related transcripts, miRNA and proteins for salt resistance

**DOI:** 10.1038/s41598-017-01360-9

**Published:** 2017-04-28

**Authors:** Guoqiang Fan, Limin Wang, Yanpeng Dong, Zhenli Zhao, Minjie Deng, Suyan Niu, Xiaoshen Zhang, Xibing Cao

**Affiliations:** 1 Institute of Paulownia, Henan Agricultural University, Zhengzhou, Henan China; 2 Zhengzhou Agriculture & Forestry Scientific Research Institute, Zhengzhou, Henan China

## Abstract

Polyploidy in plants can bestow long-term evolutionary flexibility and resistance to biotic and abiotic stresses. The upstream activation mechanisms of salt response remain unknown. Here we integrated transcriptome, miRNA and proteome data to describe the link between abscisic acid (ABA)-effectors and salt resistance against the background of Paulownia genome. Combing GO and KEGG pathway annotation of differentially expressed genes and proteins, as well as differentially expressed miRNA, these results reflect endogenous signal ABA activate the downstream effectors, such as ion channel effectors and oxido-reduction effectors, to maintain the homeostasis of Paulownia’s growth. The cascaded metabolic network involved ABA biosynthesis, signaling transduction and the response of effectors. Our results will contribute to a comprehensive understanding of the genetic basis of salt tolerance, which may help to expand the available arable land for *P*. *fortunei* cultivation.

## Introduction

The combination of low-precipitation, high-surface evaporation and weathering of native rocks accelerates naturally saline soils in arid and semi-arid climates^1^. Moreover anthropogenic activities like irrigation with brackish water and poor drainage often produce secondary salinity^2^. Soil salinity (0.2% to 0.5% soluble salt content in soil^3^) is considered as a major abiotic environmental factor that limits land utilization efficiency and affects the biomass accumulation of forests in many regions^4^. However, the genetic basis of the tolerance of forest trees to salt stress isn’t sufficiently clear despite strong demand for their cultivation in salt environments.

Tree species in the genus Paulownia are indigenous to China and are an ideal material for use in making furniture and musical instruments. Paulownia species have been incorporated into the farmland system because of their excellent characteristics and their ability to grow in various soil types and climates^5^. Furthermore, the contribution of Paulownia in improving agricultural production and facilitating were attaching human attention^6^. In our previous studies, Autotetraploid *Paulownia fortunei* obtained from its diploid via colchicine treatment has been revealed to be generally superior to its progenitor^7^. However, the underline mechanisms based on *P*. *fortunei* genome are still unknown.

The disadvantageous effects of salinity are reflected primarily by oxidative stress, osmotic stress and ion toxicity^8–11^. The formation of oxido reductase and the extrusion of Na^+^ and Cl^−^ and/or sequestration of Na^+^ and Cl^−^ into vacuoles are often observed in the response of plants to salt stress. Potassium channel proteins (KAT1)^12^, SOS1 type-Na^+^/H^+^ antiporter^13^, SOS2 type-a protein kinase and SOS3 type-calcium binding protein^14^ have been found to be involved in the prevention, reduction or repair of the damage caused by salt stress^15, 16^.

Superabundance of Na^+^ and Cl^−^ ions can induce the production of signaling molecules, such as abscisic acid (ABA), kinases and phosphatases^15, 17^ during chronic salt stress. A signaling cascade is realized by effectors that play vital roles in regulating plant growth in adverse environments. Previous studies have confirmed the roles of the potassium channel KAT1, SOS and Na^+^/H^+^ exchangers in maintaining cellular Na^+^ homeostasis^12, 15, 18^. Transporting Na^+^ from active organs (root parenchyma cells, leaf and shoot) that could be injured to protected position like the cortex and vacuole by plasma membrane to tonoplast might protect intracellular metabolism^19^. Na^+^ was found to be the vital factor that could cause stress damages in *Oryza sativa* ^20^ and *Hordeum vulgare* ^21^, while Cl^−^ was found to be the main cause of the harmful effects in *Citrus reticulate* ^22^ and *Glycine max* ^23^. These inorganic ions and some organic compounds (soluble carbohydrate and amino acid *et al*.) were shown to contribute to osmotic adjustment and the regulation of nutritional disorders^24^. The progress from signaling transduction to effectors need to be uncovered, which would be complementally characterized to surmount the adverse toxicity of salt in woody plants^4, 25^.

Salt-induced changes in mRNA and microRNA (miRNA) expression profiles, and protein abundances had been studied separately^26–28^. However, they could not depict the picture of the mechanisms of salt resistance entirely. In this study, we combined transcriptome sequencing, small RNA (sRNA) sequencing, degradome sequencing and iTRAQ technologies to deeply understand the mechanisms of salt resistance of autotetraploid *P*. *fortunei*. The roles of miRNAs in regulating their target mRNAs as well as affecting protein abundance were explored, which established the relationships among miRNA, mRNA and protein. According to the data of different types between transcriptome and proteome, the abundant researches can be unfold with single point of comparison, the comparison of two differences, multi-point sequence comparisons and multi-point non-sequence comparison^29^. We have constructed comprehensive web-based databases based on the completed whole genome sequence of *P*. *fortunei* (http://paulownia.genomics.cn/page/species/index.jsp) to illuminate the molecular mechanisms involved in salt tolerance and facilitate the molecular breeding of Paulownia. Genetic improvement of salt tolerance is crucial for sustainable forest biomass in saline areas.

## Results

### Transcriptome sequencing analysis

**Figure 1:**
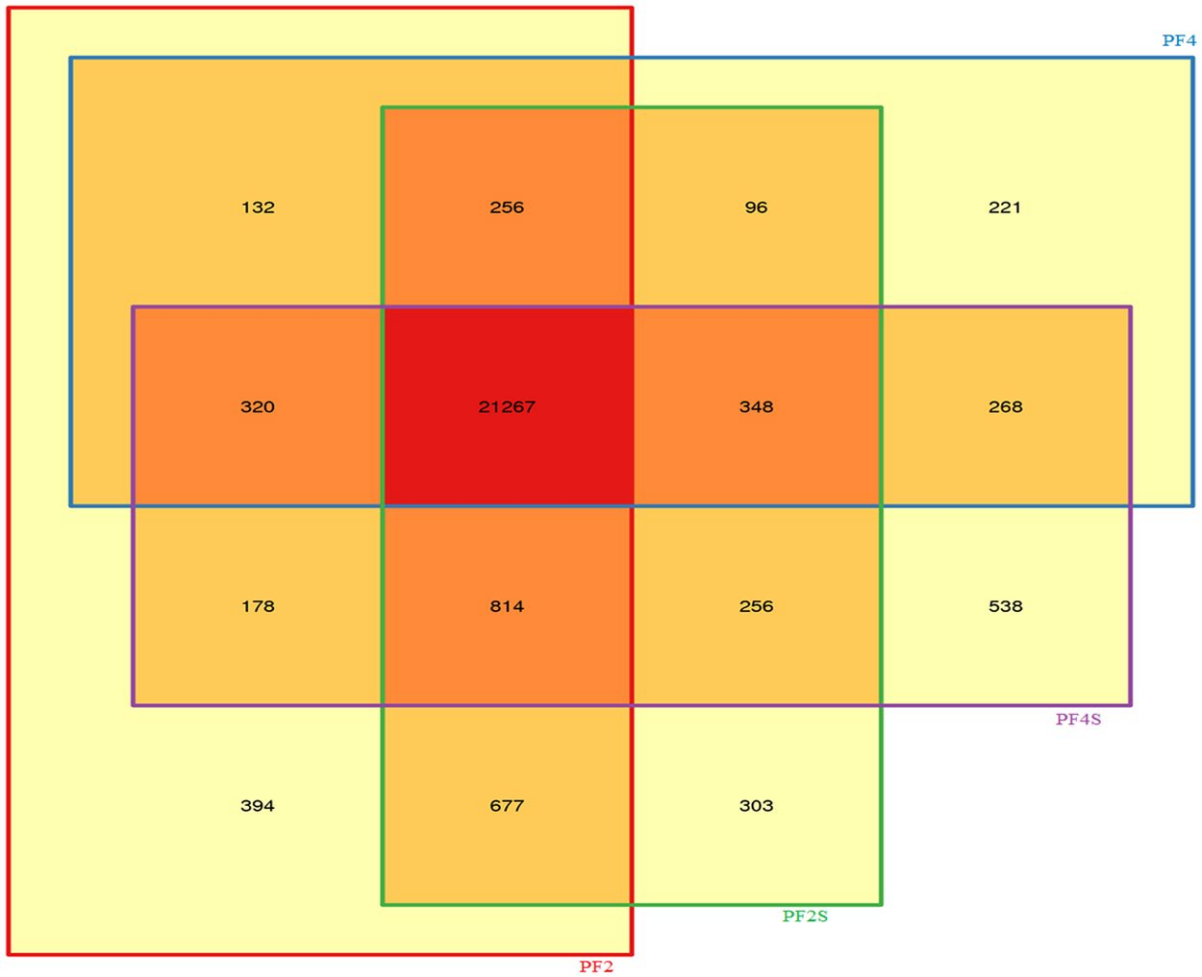
The co-expressed genes in four accessions.

**Figure 2:**
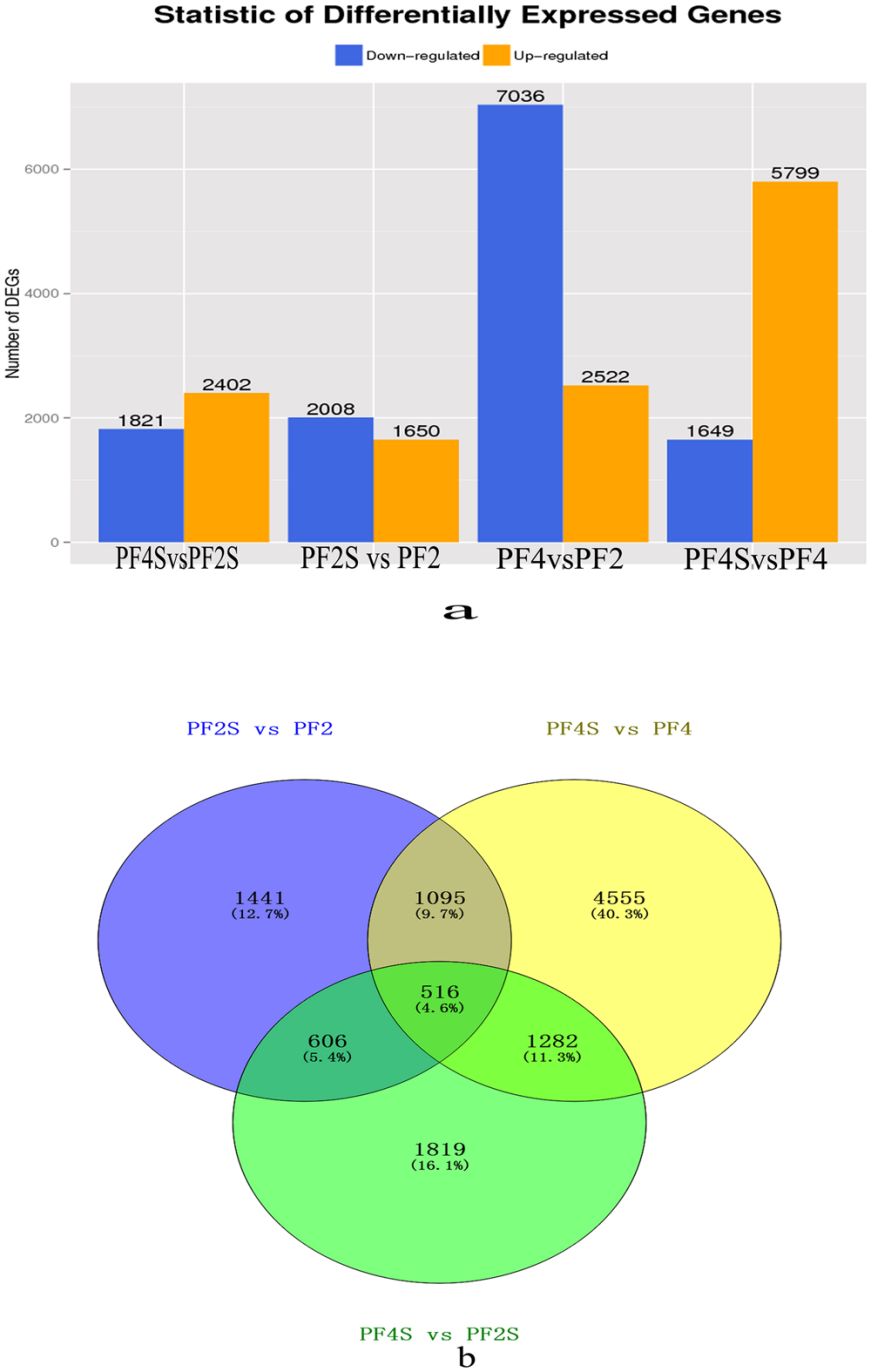
Statistics of differentially expressed genes; (**a**): The differently expressed genes in the four comparisons of PF4 *vs* PF2, PF4S *vs* PF2S, PF2S *vs* PF2 and PF4S *vs* PF4; (**b**): The overlap salt responsive genes in PF4S *vs* PF2S, PF2S *vs* PF2 and PF4S *vs* PF4.

We obtained 266,053,238 high-quality clean sequencing reads from four accessions (PF2, PF4, PF2S and PF4S), which averagely covered 79.58% of the Paulownia genome sequence and 46.45% of the Paulownia genes (Supplementary Table S1 and Supplementary Fig. S1). The gene expression analysis identified 21,267 co-expressed genes (Fig. 1), which suggested that stress and chromosome doubling induced only a fraction of specifically expressed genes. Scatter charts (Supplementary Fig. S2) of all the expressed genes in each of the libraries revealed 4223 DEGs in PF4S *vs* PF2S, 3658 DEGs in PF2S *vs* PF2, 9558 DEGs in PF4 *vs* PF2, and 7448 DEGs in PF4S *vs* PF4 (Supplementary Table S2 and Fig. 2a and b). The overlap 1611 (PF2S *vs* PF2 and PF4S *vs* PF4) DEGs indicated a general salt response.

### GO and pathway annotation analysis of DEGs

The DEGs in PF2S *vs* PF2 and PF4S *vs* PF4 were assigned to 42 and 49GO functional groups, respectively (Supplementary Fig. S3). Interestingly, the GO classifications for the DEGs correlated well with the Nr annotation for the identified DEGs. GO term thinning identified 18 and 14 significantly enriched GO terms in the PF2S *vs* PF2 and PF4S *vs* PF4 comparisons (Supplementary Table S3). The differences in these enriched GO terms indicted that they might be associated with different ploidy responses to salt stress. The DEGs were also mapped to123 (PF2S *vs* PF2) and 126 (PF4S *vs* PF4) (Supplementary Table S4) KEGG pathways. The top enrichment pathways in PF2S *vs* PF2 included “Protein processing in endoplasmic reticulum”, “Plant hormone signal transduction”, “Flavone and flavonol biosynthesis”, “Plant-pathogen interaction”, “Starch and sucrose metabolism”, “Glycerolipid metabolism”, “Sesquiterpenoid and triterpenoid biosynthesis”, “Glucosinolate biosynthesis” and “Tryptophan metabolism”. The top 14 pathways in PF4S *vs* PF4 were enriched for “Photosynthesis – antenna proteins”, “Ribosome biogenesis in eukaryotes”, “Arginine and proline metabolism”, “Galactose metabolism”, “Riboflavin metabolism”, “Synthesis and degradation of ketone bodies”, “Glycosaminoglycan degradation”, “Fatty acid metabolism”, followed by “Starch and sucrose metabolism”, “Steroid biosynthesis”, “Ascorbate and aldarate metabolism”, “Biosynthesis of unsaturated fatty acids”, “ABC transporters”, “Peroxisome”, “Glutathione metabolism”, “Oxidative phosphorylation” and “Plant hormone signal transduction”. These enriched pathways in response to salt may primarily be involved in osmotic and oxidation adjustment and nutrient balance regulated by signaling transduction.

In addition, 412 DEGs coding 44 transcription factor (TF) families in PF2S *vs* PF2 and 616 DEGs coding 48 TF families in PF4S *vs* PF4 were identified (Supplementary Table S5), respectively. TFs can act as effective effectors in the signal transduction system. The overlapped TF families in the two comparisons may reflect common salt response and universal regulation in *P*. *fortunei*.

### Salt responsive transcripts

To identify genes contributing to autotetraploid superiority in response to salt, the common 283 unique DEGs crossing PF2S *vs* PF2, PF4S *vs* PF4 and PF4S *vs* PF2S were selected (Supplementary Table S6). Transporters and channel proteins belonging to the solute carrier families together with osmotic regulation substances such as proline, soluble sugar, and dehydrin were included, which may help to regulate ion and osmotic homeostasis extra and intra the cell. Besides the identified peroxidase 3 (POD3) and polyphenol oxidase (PPO) eliminated reactive oxygen species (ROS) that involving in maintaining oxidative balance and the effectors like bHLH, NAC and MYB TFs regulating the expression of salt responsive genes were also identified.

Salt responses in plants are triggered by a series of signaling cascade reaction (Table 1). In the “Plant hormone signal transduction” pathway, ABA responsive element binding factor (ABF) acted as an ABA downstream effector that positively switched on stress response programs^30^. ABF was up-regulated in PF2S *vs* PF2 and PF4S *vs* PF4. Pyrabactin resistance protein (PYL8) acting as ABA receptor and protein phosphatase 2C (PP2C) acting as central component in ABA signal transduction were also showed up-regulated in these two comparisons. The DEG coding ABA 8′-hydroxylase (CYP707A2) that mainly reduced plant endogenous ABA showed higher expression in PF2S *vs* PF2 than in PF4S *vs* PF4. Abscisic-aldehyde oxidase (AAO), xanthoxin dehydrogenase (ABA2) and (+)-ABA 8′-hydroxylase associated with ABA synthesis were up-regulated after salt treatment. Other signaling transduction components, such as calcium ions and kinases that served as secondary messengers, were also identified. Calcineurin B protein was up-regulated in PF4S *vs* PF2S and PF4S *vs* PF4, but down-regulated in PF2S *vs* PF2. This response may initiate a signaling cascade, resulting in plant adaptive responses.

**Table 1:** The key genes in Plant hormone (ABA) signal transduction.

Gene ID	log2 Ratio (PF4S/PF4)	log2 Ratio (PF2S/PF2)	Description
PAU005576.1	1.576096	1.639984	ABA responsive element binding factor
PAU012903.1	1.668529	1.148132	abscisic acid receptor PYR/PYL family
PAU005151.1	6.394924	4.48755	protein phosphatase 2C
PAU024583.1	5.698327	4.066278	protein phosphatase 2C
PAU008363.1	1.081182	1.912569	probable protein phosphatase 2C 25
PAU024067.1	2.169048	1.861051	protein phosphatase 2C
PAU023915.1	1.683179	1.793972	probable protein phosphatase 2C 30
PAU021750.1	3.075181	1.737266	probable protein phosphatase 2C 6
PAU004222.2	3.962089	1.631191	probable protein phosphatase 2C 6 isoform 1
PAU024054.1	1.37181	1.582916	probable protein phosphatase 2C 30
PAU021138.1	3.653583	1.476211	probable protein phosphatase 2C
PAU002280.1	1.250577	1.294986	probable protein phosphatase 2C 30
PAU008956.1	1.066254	2.857354	ABA 8′-hydroxylase CYP707A2
PAU011640.1	2.625933	2.809324	CYP450 monooxygenase CYP82D33
**Calcium**			
PAU028599.1	1.606024	2.744161	Ca2+ -transporting ATPase
PAU029839.1	1.73492	2.073418	calcium-dependent protein kinase
PAU029895.1	3.3206	1.532332	calcium-dependent protein kinase
PAU007103.1	2.547659	1.008412	cation/calcium exchanger 3, solute carrier family 24 (sodium/potassium/calcium exchanger), member 6

### Pathway enrichment analysis of DEGs

**Figure 3:**
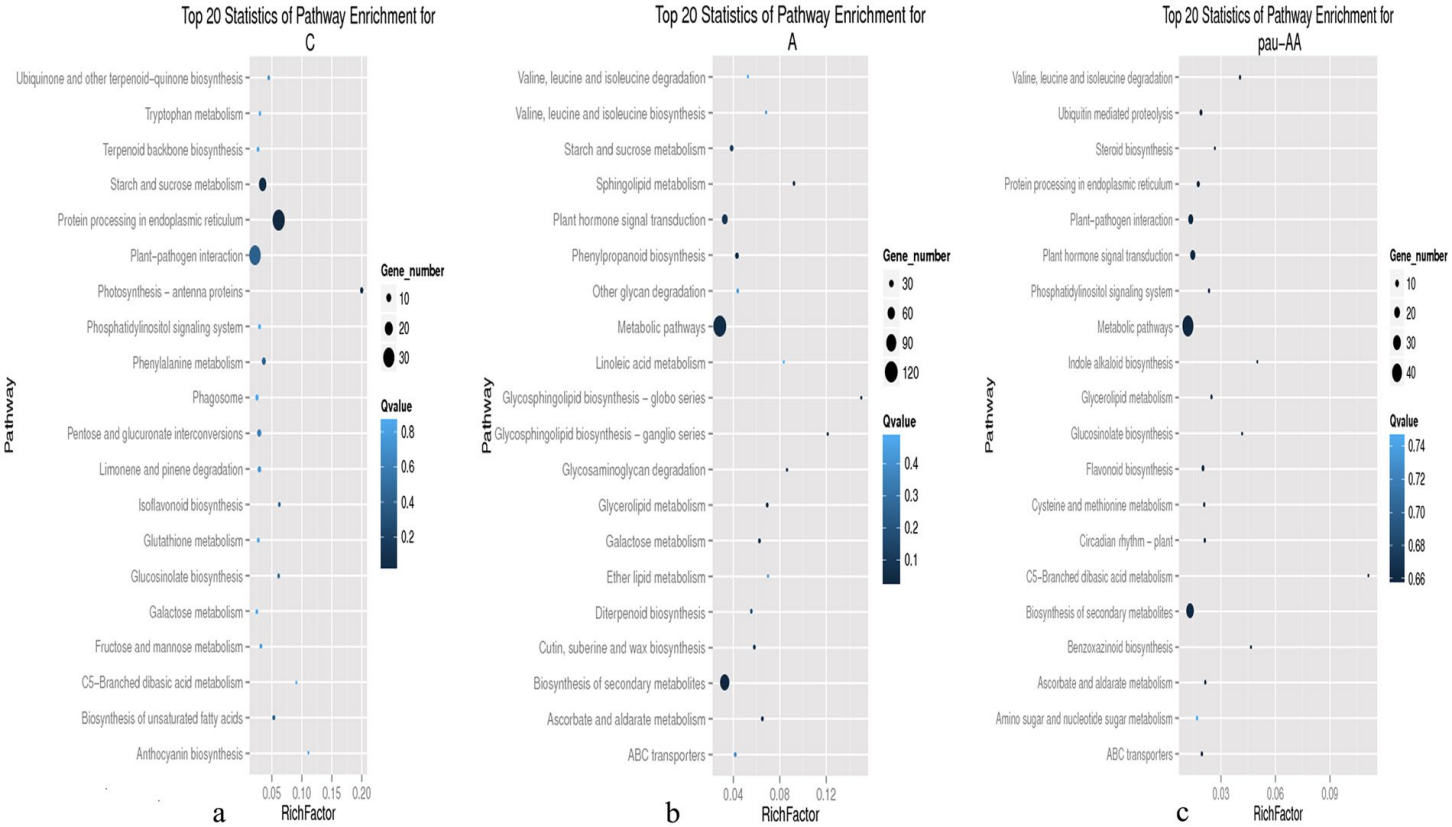
Scatter chart of top 20 pathway enrichment; (**a**): Top 20 statistics of pathway enrichment for all up-regulated genes; (**b**): Top 20 statistics of pathway enrichment for all down-regulated genes; (**c**): Top 20 statistics of pathway enrichment for Autotetraploid predominant salt response genes.

In order to illuminate the biological function of DEGs, the pathway enrichment analysis was explored. A scatter chart was obtained from the top 20 enriched pathways of all the up, and all the down regulated genes in PF4S *vs* PF2S, PF4S *vs* PF4 and PF2S *vs* PF2 (Fig. 3). The up-regulated genes were mainly enriched in six KEGG classes: Lipid metabolism; Carbohydrate metabolism; Biosynthesis of other secondary metabolites; Glycan biosynthesis and metabolism; Signal transduction; and Metabolism of terpenoids and polyketides. The down-regulated genes were enriched mainly in five KEGG categories: Folding, sorting and degradation; Energy metabolism; Photosynthesis; Amino acid metabolism; and Environmental adaptation. The predominant salt response genes (differentially expressed in PF4S *vs* PF4 and PF2S *vs* PF2, and up-regulation in PF4S *vs* PF2S) in the autotetraploid were enriched into 67 pathways (Supplementary Table S7). Some enriched pathways were involved mainly in osmotic regulation, including “Proline metabolism”, “ABC transporters” and “Starch and sucrose metabolism”; some were associated with oxidative balance regulation, including “Glutathione metabolism”, “Peroxisome”, “Oxidative phosphorylation”, and “Ascorbate and aldarate metabolism”; and some were related to signaling regulation and effector response, such as “Carotenoid biosynthesis”, “Inositol phosphate metabolism”, “Flavonoid biosynthesis”, “Plant hormone signal transduction”, “Ubiquitin mediated proteolysis” and “Oxidative phosphorylation”. “Biosynthesis of unsaturated fatty acids”, which may increase the stability of cytomembrane in salt stress environment, was also enriched.

### Quantitative analysis of the proteome of *P*. *fortunei* under salt stress

**Figure 4:**
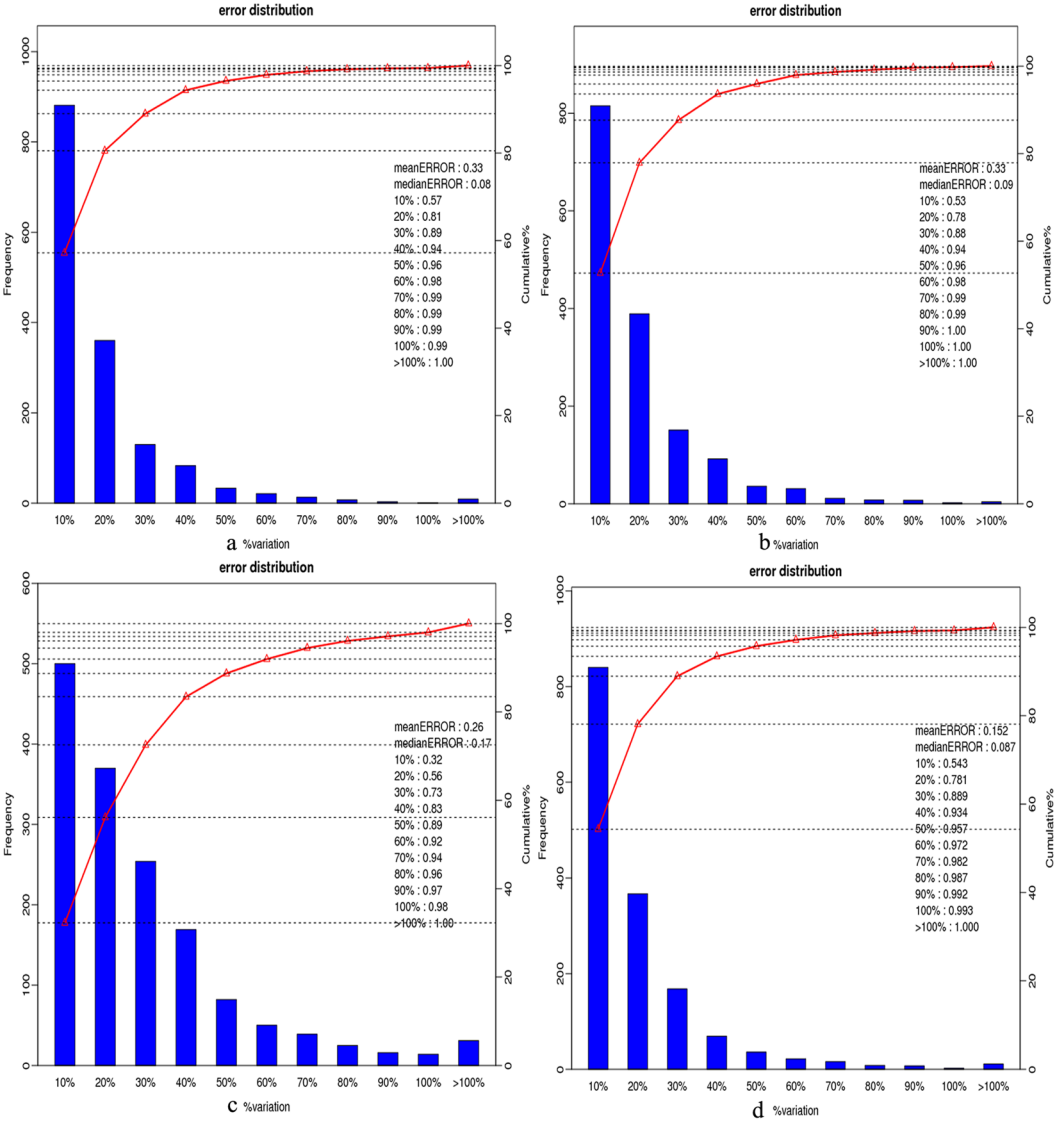
Analysis chart of repeatability in four accessions (**a**): error_PF2_2_117-VS-PF2_1_113.set1; (**b**): error_PF2S_2_118-VS-PF2S_1_114.set1; (**c**): error_PF4_2_119-VS-PF4_1_115.set1; (**d**): error_PF4S_2_121-VS-PF4S_1_116.set1).

To evaluate the effects of salt treatment on translation levels, total proteins isolating from seedling leaves of PF2, PF4, PF2S, PF4S, PF2-2, PF2S-2, PF4-2 and PF4S-2 were subjected to quantitative proteomics analysis by iTRAQ. Overall, 312,926 secondary spectrum diagrams were obtained from the extracted proteins. Among them, 34,443 spectra were matched to known spectra by Mascot software, and 23,716 unique spectra were aligned to 6891 unique peptides. To exclude possible contaminants, all proteins were confirmed with mRNA through RNA-Seq. Finally, 2486unequivocally proteins were identified. Most of these proteins were either 20–70 kDaor >100 kDa; 10–13 amino acids peptides were the most abundant; Besides, approximately 5% of the proteins had 40–60% peptide sequence coverage (Supplementary Fig. S4). Global distribution of iTRAQ ratios and the coefficient of variance (CV) showed a close correlation between replicates (CVs 0.33, 0.33, 0.152 and 0.26 for PF2S, PF2, PF4S and PF4 samples in Fig. 4, respectively). These results suggested that the proteomics analyses were reliable.

### COG, GO and KEGG pathway annotation of the proteins

To carry out functional analysis, all proteins were mapped to 24 categoriesin the COG database (Supplementary Fig. S5a and Supplementary Table S8a). “General function prediction only” was the most represented, followed by “Posttranslational modification, protein turnover, chaperones” and “Energy production and conversion”. All proteins were assigned to 47 GO terms (Supplementary Fig. S5b), among which, the most represented groups were consistent with the GO terms assigned of DEGs. The annotated proteins were mapped to 122 KEGG pathways (Supplementary Table S8b). “Metabolic pathways” (698, 28.08%) was distinctly higher than the other pathways, followed by “Biosynthesis of secondary metabolites” (381, 15.33%), “Ribosome” (127, 5.11%), “Plant hormone signal transduction”, “Starch and sucrose metabolism”, “Oxidative phosphorylation”, “Glutathione metabolism” and “Peroxisome”. The KEGG results showed that many of the mapped proteins maybe involved in osmotic adjustment, oxidation balance adjustment and signaling transduction.

### Analysis of differentially abundant proteins related to salt response

Proteins with *P*-value ≤ 0.05 and |relative abundance| ≥ 1.2 in both biological replicates were regarded as statistically significant and reproducible differences in PF2S *vs* PF2, PF4S *vs* PF4 and PF4S *vs* PF2S. A total of 678 DAPs were detected from PF4S *vs* PF2S, 604 DAPs in PF2S *vs* PF2 and 485 DAPs in PF4S *vs* PF4 were detected. Overlapping DAPs suggested a general salt response in *P*. *fortunei*.

**Figure 5:**
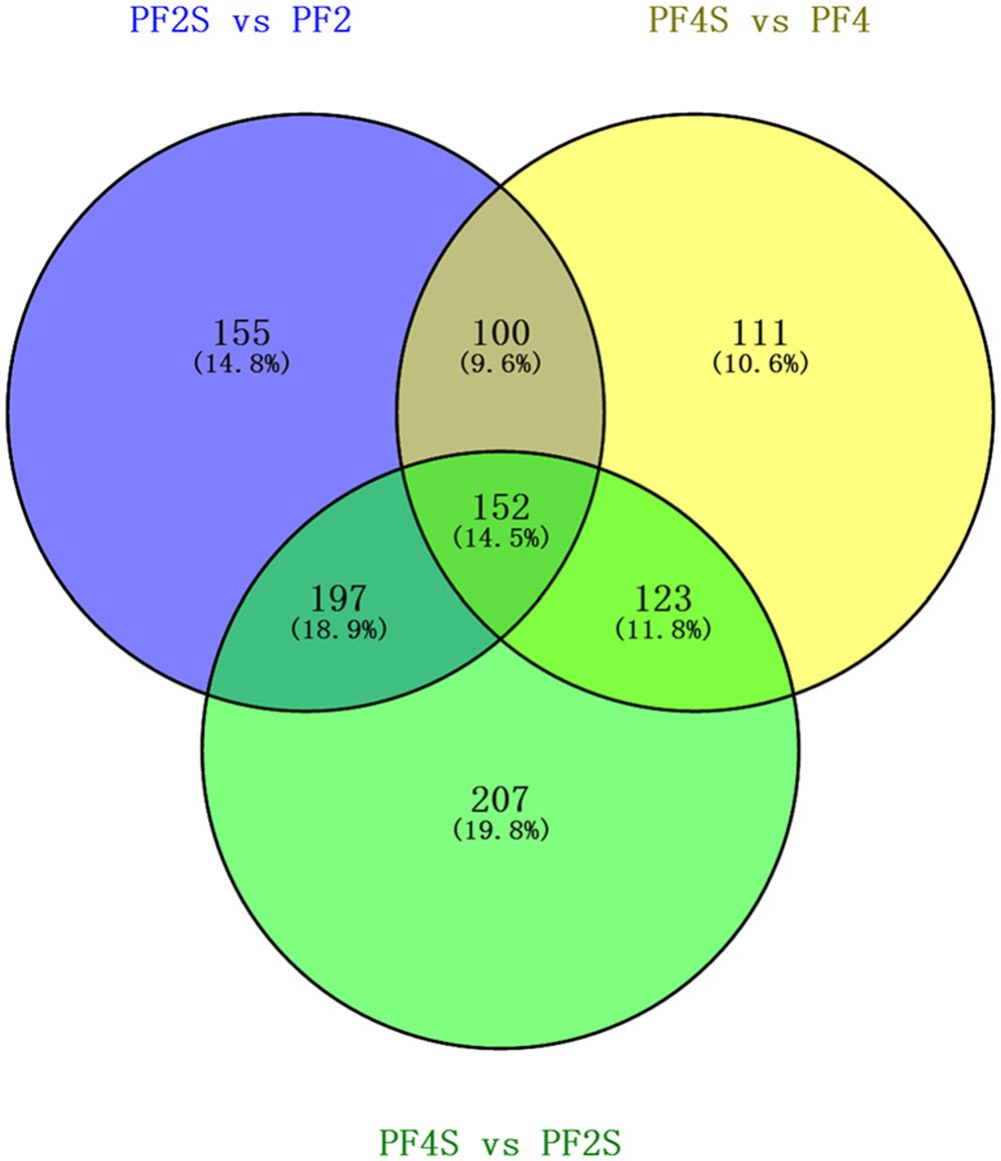
The coincidently identified differentially abundant proteins in PF4S *vs* PF2S, PF2S *vs* PF2 and PF4S *vs* PF4.

We identified 152 DAPs that were coincidently identified in PF4S *vs* PF2S, PF2S *vs* PF2 and PF4S *vs* PF4 (Fig. 5 and Supplementary Table S9 and Fig. S6). Among them 76 DAPs (up-regulation in PF4S *vs* PF2S, and differentially abundant in PF2S *vs* PF2 and PF4S *vs* PF4) were regarded involved in the salt response in autotetraploid *P*. *fortunei*. These key DAPs mainly annotated into the COG category of “Energy production and conversion” “Posttranslational modification, protein turnover, chaperones and proteins formation” and “Signaling transduction”. The most significant cellular functions and location of 76 DAPs were the same as those assigned to the modulated transcripts. Biological function prediction results indicated that some DAPs were related to signaling transduction; some DAPs were associated with osmotic adjustment, such as transporter proteins and nucleolin (Ncl), the key enzyme of soluble sugar and proteins; and some DAPs, such as PPO, POD, ferredoxin, glutathione reductase (GR), the quinone oxidoreductase (QORL1) and the monodehydroascorbate reductase (MDAR5), were involved in oxidative balance by eliminating ROS.

Among the key 76 DAPs, 72.4% were annotated into significant GO enrichment terms (Supplementary Table S10a). Membrane system was an enriched term indicating that it may be involved in plant signal transduction and transmembrane transport of ions. The enriched ATPase activity and enzyme activity terms indicated that they may be related to the synthesis of carbohydrates and lipids, and involved in maintaining intracellular osmotic balance and normal energy. Additionally, some DAPs were primarily enriched into terms related to stimulation response.

The down-regulated proteins were enriched mainly in photosynthesis, and the up-regulated proteins were primarily enriched in energy metabolism, carbohydrate metabolism, amino acid metabolism, and secondary metabolites, which was similar to the KEGG annotation of DEGs (Supplementary Table S10b). Some proteins, pathogenesis-related PR-1, the abscisic acid receptor PYR/PYL family and serine/threonine-protein kinase SRK2, happened to up-regulated in “Plant hormone signal transduction”.

### Small RNA sequencing analysis

To understand the post-transcriptional modification of the differentially expressed mRNAs caused by miRNAs, small RNA sequencing analysis were conducted. MiRNA was one of important parts to affect the stability and translation efficiency of mRNAs.

MiRNA sequencing results indicated that the rate of clean reads was 98.9%. Reads of 21–24 nt were the most abundance, and the number of 21–22 ntsRNAs increased after salt stresss (Supplementary Fig. S7a). After aligning the reads with the Paulownia genome sequence, 2,059,182 (PF4), 2,438,296 (PF4S), 3,148,132 (PF2) and 3,207,267 (PF2S) sRNA were identified. A total of 45,375,903 clean reads mapped to the Paulownia genome and were distributed across the different chromosomes (Supplementary Table S11 and Fig. S7b).

### Identification of known and novel miRNAs

We identified 134 conserved miRNA (including 127 contained *sequences) which belonged to 24 families and 183 novel miRNAs (101 of which had corresponding miRNA *sequences) (Supplementary Table S12). These miRNAs varied from 20–26 nt in length, with 39.10% being 21-nt long. The minimum free energy (MFE) of the pre-miRNA structures ranged from −18.00 to −153.40 kcal/mol. The most abundant miRNA family was MIR166 (containing 15 members), most of which were located close together, followed by the MIR156 and MIR171-1 families (both containing 13 members). These three families were known to berelated with abiotic stress^31–33^.

### Salt responsive miRNAs in *P*. *fortunei*

**Figure 6:**
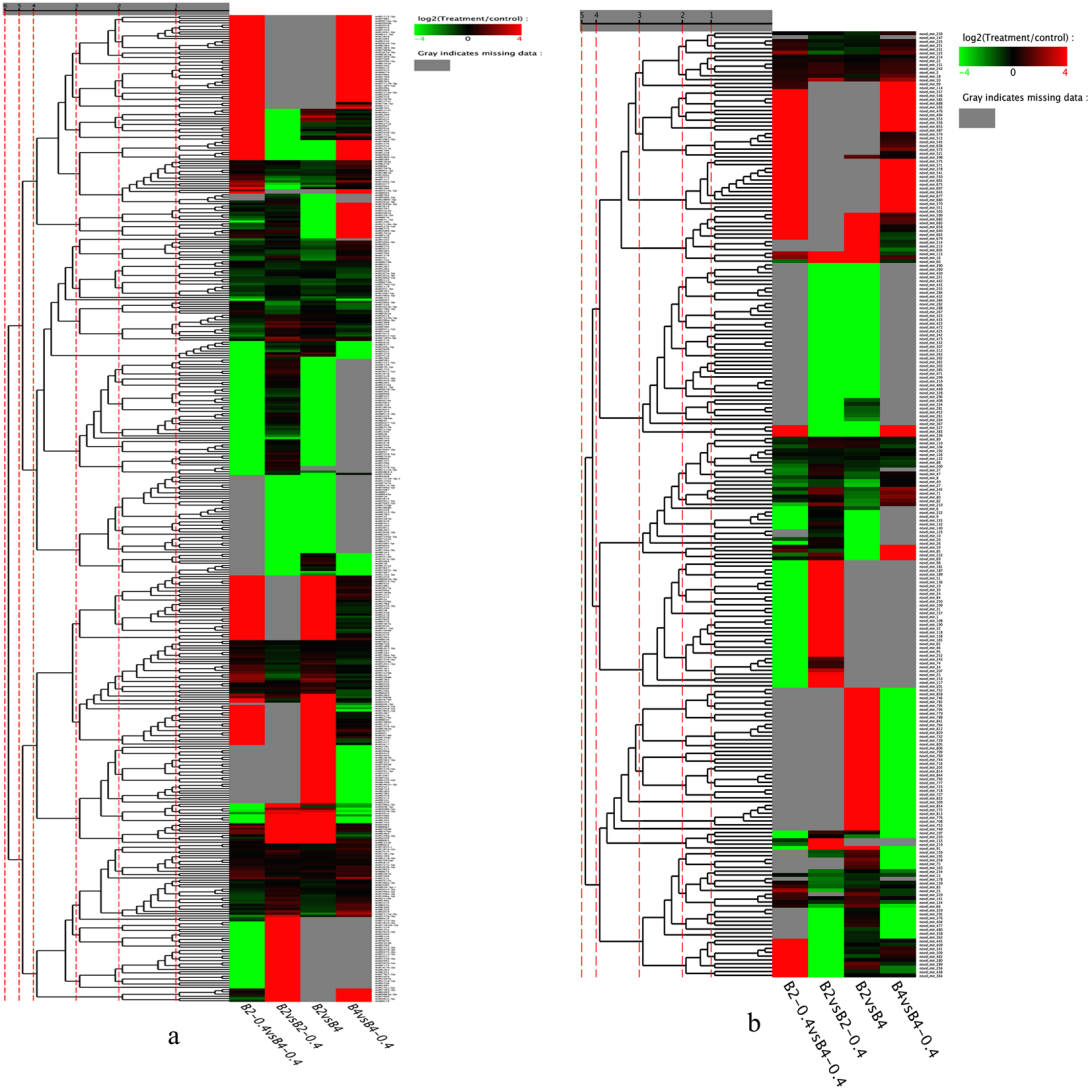
The cluster chart of miRNAs’ expression; (**a**): Conserved miRNAs; (**b**): Novel miRNAs.

We selected miRNAs with total expression abundance >10 for further analysis. A total of 193miRNAs (*P* < 0.05 and |fold change| ≥ 1) in the four libraries were identified as differentially expressed, including 78 known miRNAs and 115 novel miRNAs (Fig. 6 and Supplementary Table S13). Most members of the same miRNA family had similar differential expression patterns.

Three conserved miRNAs (pfo-miR167i, pfo-miR166d and pfo-miR398d) were consistently down-regulated (−11to −2.5 fold changes) in the PF2S *vs* PF2 and PF4S *vs* PF4 comparisons. Eight conserved miRNAs (pfo-miR167a/c/d, pfo-miR408a, pfo-miR159, pfo-miR172d, pfo-miR393c and pfo-miR390a) were down-regulated only in PF4S *vs* PF4 but not in PF2S *vs* PF2. Additionally, pfo-miR319c and pfo-miR398b/c showed opposite expressed trends between PF2S *vs* PF2 (up-regulated) and PF4S *vs* PF4 (down-regulated). Pfo-miR171k/l were up-regulated after salt stress but the level of up-regulation was lower in PF4S *vs* PF4 compared with PF2S *vs* PF2. All of these differentially expressed miRNAs (Table 2) belonged to nine miRNA families (MIR167-1, MIR166, MIR398, MIR408, MIR159, MIR172, MIR393, MIR390 and MIR171_1), suggesting that members of these families may play vital roles in the post-transcriptional regulation of mRNAs involved in salt response of Paulownia. Among the novel miRNAs, 13 were down-regulated significantly in PF4S *vs* PF4 and less in PF2S *vs* PF2 (Table 3).

**Table 2:** The information of some differentially expressed miRNA.

mirNA	Family ID	Fold-change (log2 ratio)		PF4S/PF4	p value	FDR
		PF4S/PF2S	PF2S/PF2			
pfo-miR167i	MIR167-1	0	−10.1669556	−10.7628095	1.13E-05	1.77E-05
pfo-miR166d	MIR166	0	−9.34098500	−9.14429447	1.39E-20	2.36E-12
pfo-miR166d	MIR398	5.8760719	−5.55962529	−2.50268782	5.84E-10	9.81E-10
Pfo-miR167a	MIR167_1	−17.79471147	0.697989138	−16.0599278	0	0
Pfo-miR408a	MIR408	0	0	−15.4696131	0	0
Pfo-miR159	MIR159	0	0	−14.1104860	0	0
Pfo-miR172d	MIR172	−10.5708353	0.593357552	−8.34961337	3.94E-14	6.98E-14
Pfo-miR393c	MIR393	0	0	−8.19433515	3.40E-15	6.65E-15
Pfo-miR390a	MIR390	−4.17019555	0.17635398	−1.75883584	8.11E-13	1.42E-12
Pfo-miR167c	MIR167_1	−0.92700548	−0.2728505	−1.10977026	8.34E-169	2.80E-168
Pfo-miR167d	MIR167_1	−0.92700548	−0.2728505	−1.10977026	8.34E-169	2.80E-168
Pfo-miR319c	MIR159	−8.14322918	8.14322918	−6.73490353	5.27E-06	8.52E-06
Pfo-miR398b	MIR398	−6.09116133	5.77825132	−2.44754627	1.14E-05	1.77E-05
Pfo-miR398c	MIR398	−6.09116133	5.77825132	−2.44754627	1.14E-05	1.77E-05
Pfo-miR171k	MIR171_1	−1.98861659	3.31263074	11.80250268	2.33E-67	6.63E-67
Pfo-miR171l	MIR171_1	−1.98861659	3.31263074	11.80250268	2.33E-67	6.63E-67
Pfo_mir_122a	NON	9.319866034	0	−9.31986603	2.36E-26	6.25E-26
Pfo_mir_136b	NON	10.89981046	0	−10.8998104	2.06E-08	3.91E-08
Pfo_mir_136a	NON	9.319866034	7.62865601	−9.31986603	2.91E-32	8.30E-32
Pfo_mir_225d	NON	10.75171182	0	−10.7517118	2.06E-08	3.91E-08
Pfo_mir_245a	NON	10.25846549	0	−10.2584654	2.98E-54	1.36E-53
Pfo_mir_718	NON	10.40023945	0	−10.4002394	2.22E-67	1.20E-66
Pfo_mir_724	NON	9.274062345	0	−9.27406234	2.36E-26	6.25E-26
Pfo_mir_757	NON	10.93457588	0	−10.9345758	7.27E-08	1.36E-07
Pfo_mir_761	NON	10.59908968	0	−10.5990896	3.22E-09	6.32E-09
Pfo_mir_792	NON	10.53580343	0	−10.5358034	1.65E-09	3.36E-09
Pfo_mir_799	NON	10.59288453	0	−10.5928845	3.22E-09	6.32E-09
Pfo_mir_806	NON	10.56145202	0	−10.56145202	3.22E-09	6.32E-09

**Table 3:** The annotation of 12DEGs using qRT-PCR analysis.

Gene ID	Description
PAU024715.1	ABC transporter I family member 6
PAU013189.1	calcium ion binding protein
PAU025334.3	potassium channel KAT1
PAU000825.1	transcription factor bHLH143
PAU009169.1	major latex-like protein
PAU024054.1	probable protein phosphatase 2C 30
PAU027327.1	glutathione S-transferase
PAU018833.1	bacterial-induced class III peroxidase
PAU013408.2	amino acid permease 2, solute carrier family 32
PAU029564.1	polyphenol oxidase
PAU007545.1	heat shock cognate protein 80
PAU018567.1	Ras-related protein Rab-6A

### Predicted target genes of the conserved and novel miRNA by degradome sequencing

To predict the target genes of the identified miRNAs, degradome sequencing was conducted. After filtering, 84.28% (PF2), 84.39% (PF2S), 77.15% (PF4) and 76.26% (PF4S) clean reads were aligned to the Paulownia genome sequence (Supplementary Table S14a). The prediction results indicated that 12 target genes may be cleaved by 10 conserved miRNAs, and 79 target genes may be cleaved by 90 novel miRNAs (Supplementary Table S14b). Several target mRNAs were predicted targets of more than one unique miRNA, while several miRNAs belonging to different miRNA families were predicted to target the same genes. Pfo-miR8759 may target three genes (phospholipase A1, galactokinase and Lon protease 2), and pfo-M20 and pfo-M792were consistently predicted to target nuclear pore complex protein Nup62.

The predicted target genes were aligned to the Nr, GO and KEGG databases using BLAST searches. The differently expressed target genes were classified into two categories. One category was directly related to defense against salt stress such as momilactone A synthase, potassium channel KAT1, Xaa-Pro amino peptidase, MATE efflux family protein 6, isoflavone 7-O-glucosyltransferase, alpha-L-fucosidase, polyphenol oxidase, S-formyl glutathione hydrolase, S-locus glycoprotein, NADH dehydrogenase, delta (24)-sterol reductase, monodehydroascorbate reductase, 10 kDa chaperonin, potassium channel, thiamine thiazole synthase 2, mitochondrial chaperone BCS1-Aand 1-deoxyxylulose-5-phosphate synthase. The other category was indirectly related to response to salt, which were associated with regulation of gene expression and signaling transduction, such as RING finger and CHY zinc finger domain-containing protein 1, E3 ubiquitin-protein ligase RNF170, B3 domain-containing protein At2g36080, bHLH143, NAC TFs, SNF1-related protein kinase, cytokinin-regulated kinase 1, Lon protease 2, calcium ion binding protein and galactokinase. These target genes were annotated into “Phosphatidylinositol signaling system”, “Plant-pathogen interaction”, “Plant hormone signal transduction”, “Carotenoid biosynthesis”, “Starch and sucrose metabolism”, “Protein processing in endoplasmic reticulum”, “Oxidative phosphorylation”, “Peroxisome”, “Arginine and proline metabolism”, “Glutathione metabolism”, “Inositol phosphate metabolism”, and “ABC transporters”, which are associated mainly with the stress signaling cascade amplifier and stress response, and oxidation and osmosis regulation.

### Integration analysis of the transcriptome, proteome and miRNA

We compared and explored the expression levels of mRNAs and miRNAs, protein abundance and the post-transcriptional regulation of target mRNAs by miRNAs. The percentage correlation between the transcriptome sequences and proteins was less than 40%. This low correlation may be explained by the differences in experimental system and data types, as well aspost-transcriptional modification and transcriptional inhibition, and selectively expression of mRNA. Consistent expression trends between transcriptome and proteomic levels showed linear correlations, but it could not apply to different expression trends of quantitative proteins and genes (Supplementary Fig. S8).

By comparing the transcriptome and proteome data, 2437 (PF2), 2406 (PF4), 2439 (PF2S) and 2431 (PF4S) proteins were detected to encode correspondingly by mRNAs (Supplementary Table S9); however, the correlation between the protein and mRNA levels in response to salt stress was not direct. The repression of mRNA translational and/or mRNA degradation was negatively affected by miRNAs.

In PF2S *vs* PF2, 20 DEGs and six DAPs were predicted as miRNA targets, and in PF4S *vs* PF4, 34 DEGs and three DAPs were predicted as miRNA targets (supplementary Table S15). The differentially expressed pfo-miR171b regulated its target gene coding calcium ion binding protein. Two mRNA-protein pairs, the *PPO* gene and major latex-like protein, showed consistently high expression and abundance. *PPO* was the target of pfo-mir348, but pfo-mir348 was not differentially expressed in the three comparisons. Meanwhile, two mRNA–miRNA targets, KAT1 and alpha-L-fucosidase, showed negative correlation among pfo-miR398, pfo-mir255 and their target mRNAs. Alpha-L-fucosidase also showed high abundance in its protein profile. These key genes and proteins were annotated as “Glycan biosynthesis and metabolism”, “Chaperones and folding catalysts” and “Oxidoreductases”, which are primarily involved inreducing stress ions and maintaining homeostasis of the intracellular oxidation environment.

### QRT-PCR analysis

**Figure 7:**
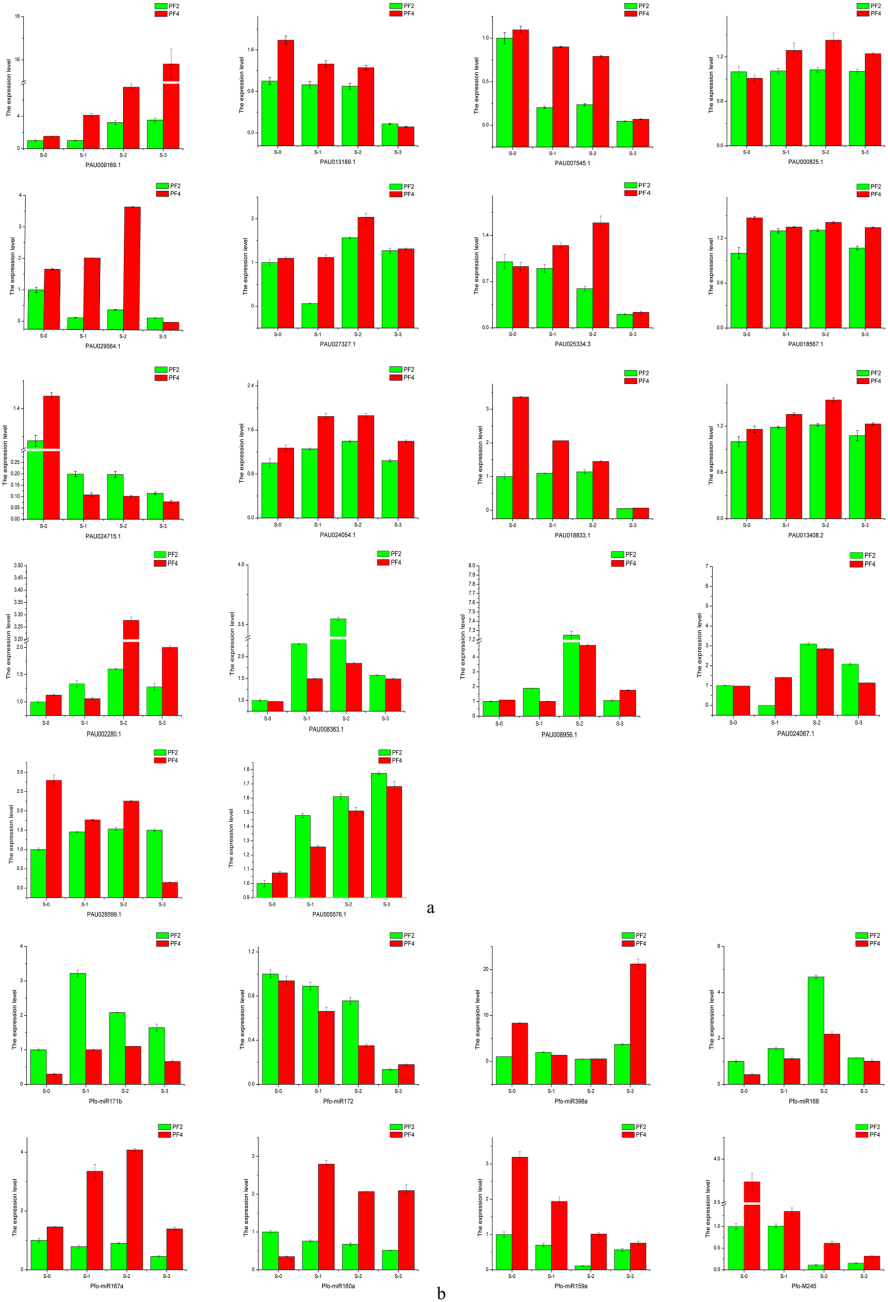
Quantitative Real-Time PCR (qRT-PCR) analysis of 18 selected differentially expressed genes (**a**) and 8 differentially expressed miRNA (**b**). 18S rRNA was used as the internal reference gene. For each group, the PF2 (S-0) expression level was considered as 1.00, and other samples were normalized accordingly. Standard error of the mean for three technical replicates is represented by the error bars. S-0, 0 day 70 mM salt-treated for PF2 and PF4; S-10, 10 days 70 mM salt-treated for PF2 and PF4; S-15, 15 days 70 mM salt-treated for PF2 and PF4; S-20, 20 days 70 mM salt-treated for PF2 and PF4.

To confirm the reliability of sequencing technology, 18 DEGs (Table 3) and 8 miRNAs (selected from Table S12) of different stress stages induced by 70 mM NaCl were selected for qRT-PCR analysis (Fig. 7). Among them, 12 DEGs were randomly selected from differentially expressed genes; the other DEGs were specially selected from the pathways of ABA signaling transduction. Results indicated that 17 genes and 8 miRNAs were almost consistent between the qRT-PCR and sequencing analyses at S-2 sites (15d salt stress). The selected genes were involved in positively salt response, such as redox homeostasis (glutathione S-transferase, bacterial-induced class III peroxidase and polyphenol oxidase), osmotic balance (ABC transporter I family member 6, heat shock cognate protein, major latex-like protein and solute carrier family 32), intracellular ion homeostasis (potassium channel KAT1, ABC transporter I family member 6 and solute carrier family 32), plant hormones (ABA) signaling and regulation pathway (calcium ion binding protein, transcription factor bHLH143, probable protein phosphatase 2 C/25/30, Ras-related protein Rab-6A, ABA responsive element binding factor, ABA 8′-hydroxylase CYP707A2 and Ca2+ -transporting ATPase). Among them, almost all of the expression levels at S-3 (20 salt treatment) displayed lower, which lead to the non-linear trend in different salt stress stages, suggesting that 20 days treatment might be close to the salt endurance limit of Paulounia seedlings. However, this did not rule out the existence of errors, such as the S-1 of PAU024067.1 in PF2. Additionally, the miRNAs (Pfo-M245, Pfo-miR171b and Pfo-miR398a) and their target genes (transcription factor bHLH143, calcium ion binding protein, potassium channel KAT1) all showed opposite expression trends, which confirmed the cleavage function of the corresponding miRNAs. Besides, Pfo-miR159a and its target genes displayed consistent expression trend, which might due to the translational inhibition of miRNA. The inconsistency trends, such as rise first then fall, were probably more sensitive in the detection of low abundant transcripts and small changes in gene expression of sequencing than qRT-PCR method. In all, these results indicated the validity of the sequencing method.

## Discussion

The availability of genome sequences helps to promote the study of non-model plants. The quality of the reference genome sequences is important in fully utilizing the biological information they contain. Based on Paulownia genomes, the key genes, and/or proteins and/or miRNA of interest^34^ can be more accurate comparing with the transcriptome background^6, 35, 36^. Besides the availability of the genome information is helpful for comparative genomics studies among different plant species, the valuable insights into genome reorganization, and the evolution of gene families^35^. It is a general research to reveal salt resistance mechanism^37^ and water resistance mechanism^38^ using transcriptome, miRNA or proteome analysis. However, researches involving the integration of transcriptome, miRNA, degradome sequencing and proteome technologies are rare.

Polyploidization determines the possible evolution of postzygotic reproductive isolation, which drives changes in ecological tolerance and the competitive situation, and expands the areas that the plant can inhabit, as well as increases the biodiversity and genetic polymorphism^39–41^. Epigenetic phenomena of polyploidy, a form of evolutionary potential, can increase the diversity and plasticity of species, which promotes adaptive potential and abiotic stress resistance^42^. Results of this study indicated that there were more genes down-regulated than up-regulated in PF4 *vs* PF2, when threatened by salinity, while many genes show up-regulated in PF4S *vs* PF4. Because of the dosage effect of genes, many genes were down-regulated under usual environment to stay in a normal level. Once stressed, there if a possibility that some of these genes may be up-regulated to maintain homeostasis. Polyploids general form a novel physiological and life-history characteristics absent in the progenitor cytotype^43^. Therefore Polyploid plants universally have the advantage over diploid plants in their responses to abiotic stresses.

The integration of several technologies inevitably exist differences in the background. High correlation coefficients were observed in same and/orcontrary expressional tendencies between proteins and genes (r ≥ 0.78). Similar to our results, up-regulated genes and protein concentrations were well correlated in yeast^44^. The squared Pearson correlation coefficient was low between the quantitative proteins and genes (r < 0.50). Vogel (2012) declared that the correlation between mRNAs and proteins from various organisms were not strong^45^. More report claimed that entire non-correlation existed in bacteria between abundances of proteins and mRNAs^46^. In addition to different species, the combinations of post-transcriptional regulation, post-translational modification and noise data, as well as the equilibrium between the synthesis and degradation rates of the participating molecules may response for this^45^.

Combining GO and KEGG pathway annotation of DEGs and DAPs, as well as differentially expressed miRNA, these results suggested that the endogenous signal ABA activated the downstream effectors, such as ion channel effectors andoxido-reduction effectors, in order to maintain the homeostasis of Paulownia’s growth.

Plant hormones play vital roles in regulating gene expression in plants under abiotic stresses. At the beginning, the bound ABA is released and the degradation rate of ABA is decreased, followed by ABA *de novo* synthesis, which is increased^47^. In the carotenoid biosynthesis pathway, AAO, ABA2 and (+)-ABA 8′-hydroxylase associated with ABA synthesis were up-regulated after salt treatment, which probably lead to a beneficial increase in ABA concentrations. Other up-regulated genes (PYR, PP2C and ABF) in the enriched “Plant hormone signal transduction” pathway regulate stomatal closure to decrease Paulownia transpiration, which will help to protect the plants from damaging due to insufficient water. Meanwhile, stress signals are transferred to downstream effectors. The ABA receptors PYR1/PYLs/RCARs bind ABA and downstream. The primarily negative regulator PP2Cremoves the inhibition of protein kinases, such as SnRK and CDPK, in the pathway^48^. These protein kinases can directly phosphorylate downstream target effecters such as KAT1, ABC transporter, Ncl and the soluble carrier family^49, 50^.

PfoMIR398 was down-regulated in PF4S *vs* PF4 and PF4S *vs* PF2S, but up-regulated in PF2S *vs* PF2. Its target gene, potassium channel KAT1, was present up-regulated. The Ca^2+^ content is controlled by ABA, which helps to regulate the potassium channels^51^. ABA may indirectly regulate the effector KAT to keep the homeostasis of potassium, which contribute to the enzyme activation, stabilization of protein synthesis and neutralization of negative charges on proteins by directly binding with proteins, as well as a major factor for osmotic adjustment^52^. The fragmentation of KAT1 strongly affected the growth of seedlings^53^. It has been shown that insufficient K^+^ induced the accumulation of H_2_O_2_ as well as ROS production as an early root response^54^. Indeed, the K^+^/Na^+^ ratio is considered an effective parameter for evaluating the adaptation of plants to a saline environment^55^. Additionally, the potassium channel is important for biofilm formation^56^. The conserved sequence G(262)Y(263)G(264) in the KAT1 P structural domain was reported to be involved in the alternative uptake of intracellular ions, and the mutation in this sequence decreased the permeability of K^+^ and increased the permeability of Na^+^^57^.

Oxido-reduction effectors contribute to oxidative balance and protect photosynthesis. PPO and major latex protein related to autotetraploid superiority is entirely correlation between DAPs and DEGs. PPO is a rhizospheric enzyme that facilitates the efficient utilization of the root nutrients**;** besides it is also an antioxidant enzyme that plays a key role in maintaining the homeostasis of intracellular oxidoreduction environment^58, 59^. It has been suggested that PPO catalyses the synthesis of lignin and quinone compounds, which can protect plants from damaging coming germs by forming protective shield^60^. Consistently, the activity of average PPO enhances salt response in *Ocimum basilicum* ^61^. In ABA signal transduction pathways, PPO was reported to act as an activator of downstream units, and as an intermediate effector that controls the detoxification of ROS^62^.

PPO can act as a non-destructive container that can absorb extra light energy, causing the photoreaction stage redundant. Thereby photo-oxidative damage in plants under salt stress can be alleviated^63^. Other studies have shown that high PPO levels are not necessarily linked with the ABA concentrations, and these PPOs probably belong to different signal transduction pathways^63^. However, high levels of PPO may promote the consequent activation of ABA-mediated stress responses. Under salt stress, the high levels of ABA decrease the stomatal aperture and reduce CO_2_ intake. Thus, the extra assimilatory power and the superoxide anion might threaten the stability of the photosystem^64^. Meanwhile, PPO consumes extra assimilatory power and eliminates the oxyradical, which protects the photosynthetic mechanism but does not control the decreased biomass.

## Materials and Methods

### Plant materials and stress treatments

All plantmaterials were obtained from Institute of Paulownia, Henan Agricultural University, Zhengzhou, Henan Province, China. Tissue culture seedlings were cultivated in a controlled growth chamber for 30 days with a 16-/8-h light/dark cycle at 25 ± 2 °C and a photon flux intensity of 130 μmol·m^−2^ s^−1^. The samples with uniform growth (crown size and height) were transplanted into outdoors nutritive bowl (20 cm in diameter at the bottom and 20 cm deep) with substrate for 30 days. Plants with uniform growth were selected and transferred individually into nutrition pots (30 cm in diameter at the bottom and 30 cm deep) containing normal garden soil (19 mM NaCl) with trays underneath. After 50 days, *P*. *fortunei* seedlings with consistent growth were selected and subjected to salt treatment basing on the method of Deng *et al*.^65^. Three repetitions were set up. The selected diploid (PF2) and autotetraploid (PF4) *P*. *fortunei* plants were induced from PF2 through colchicines treatment. They were treated with 0, 35, 70 and 105 mM NaCl for 15 days, respectively. Physiological measurements and morphologic observation were reported in our previous studies^6, 65^. Samples treated with 0 mM (PF2 and PF4) and 70 mM (PF2S and PF4S) NaCl were selected for the following analysis. The 70 mM NaCl treatment was chosen because it is close to the threshold tolerance of Paulownia. For the quantitative real-time polymerase chain reaction (qRT-PCR) analysis, PF2 and PF4 were treated with 0 and 70 mM NaCl for 0, 10, 15 and 20 days, respectively. The second pair of leaves were collected from the apex shoot of PF2, PF4, PF2S and PF4Sand blended separately. Three repetitions were performed in every step. The four blended leaf samples were frozen in liquid nitrogen and stored at −80 °C for total RNA extraction.

### Total RNAs extraction, RNA-seq analysis and bioinformatics analysis

Total RNAs were extracted from the PF2, PF4, PF2S and PF4S leaf samples using Trizol reagent (Invitrogen, Carlsbad, CA, USA). RNA purification was performed using an RNeasy Mini Elute Cleanup Kit (Qiagen, Dusseldorf, Germany) according to the manufacturer’s protocol. Total RNAs were treated with DNase I (TaKaRa, Dalian, China) to avoid genomic DNA contamination. Four libraries were prepared for transcriptome and degradome sequencing (IlluminaHiSeq™ 2000: Illumina, San Diego, CA, USA). Then, equal parts of the RNA samples from the four libraries were used for sRNA sequencing.

The sequencing libraries for transcriptome analysis were prepared according to Illumina’s kit (Illumina, San Diego, CA, USA). The synthesis of cDNA referred to the previous reported^6^. The short cDNA fragments were then connected to adapter sequences. After agarose gel electrophoresis, suitable fragments were selected as templates for PCR amplification as templates.

An Agilent 2100 Bioanalyzer (Agilent Technologies, Palo Alto, CA, USA) and an ABI Step One Plus Real-Time PCR System (ABI, New York, NY, USA) were used to quantify and quality check the amplified products. The final products were loaded onto an Illumina HiSeq™ 2000 platform for transcriptome sequencing.

The generated raw data were filtered to obtain clean data described in our previous study^6^. The remaining clean reads were mapped to the *P*. *fortunei* genome using SOAPaligner/SOAP2^66, 67^ withno more than **f**ive mismatches. At last the genome, gene coverage and reads distribution were determined.

### Small RNA sequencing and miRNA identification

Equal amounts of the RNA in the four RNA libraries (PF2, PF4, PF2S and PF4S) were pooled to generate four sRNA libraries. Briefly, 4 μg of the RNAs, which were isolated from the 18–30 nt by polyacrylamide gel electrophoresis (PAGE) band, were successively linked with 5′ and 3′ adapters. Single-stranded cDNA was created by reverse transcription and amplified by 12 cycles of PCR. The cDNA was purified by PAGE then dissolved in EB solution and sequenced on aIllumina HiSeq™ 2000 platform at BGI (Shenzhen, China).

The raw RNA reads were processed to filter low quality reads, as described previously^68^. SOAP^69^ was used to map the sRNAs to the Paulownia genome allowing no more than two mismatches to determine their expression and distribution on the genome allowing less than 2 mismatches was adopted to map the small RNA to Paulownia genome.

The sRNA read sequences were searched against the Genbank (ftp://ftp.ncbi.nlm.nih.gov/genbank/) and Rfam databases (http://rfam.janelia.org/, Version: 11.0) using BLASTN (http://blast.ncbi.nlm.nih.gov/Blast.cgi?PROGRAM=blastn) and matched sequences were removed from the sRNA dataset (Release 10)^70^. The remaining sRNAs were aligned with the plant mature miRNAs in miRBase (http://www.mirbase.org/ftp.shtml, Release 21.0) and mapped to the Paulownia genome to identify conserved miRNAs with no more than two mismatches.

The unannotated sRNAs that did not map to the genome were input to mfold (http://mfold.rna.albany.edu/?q0mfold) and Mireap (https://sourceforge.net/projects/mireap/) to identify potential novel miRNAs by exploring their secondary structure, dicer cleavage site and minimum free energy^71^. The hairpin structure of miRNA precursors also can be used to predict novel miRNAs. The criteria used to define novel miRNA have been described previously^72^.

### Degradome sequencing and Identification of miRNA targets

Four degradome libraries corresponding to PF2, PF4, PF2S and PF4S were constructed as described previously^73^. RNAs with poly (A) in the 3′adapter and a *Mme*I (NEB, Ipswich, MA, USA) recognition site in the 5′ adapter were isolated. The cDNA libraries construction method is consistent with the method described by Niu *et al*.^74^. The final cDNA library was purified and sequenced using an Illumina HiSeq™ 2000 system (Beijing Genomics Institute, Shenzhen, China). The degradome sequencing reads with 20 and 21 nt were used in the PairFinder software to identify potentially cleaved targets^75^.

The degradome reads were align to the unique sequence signatures of the Paulownia genome using SOAP. The aligned sequences were extended to 31 nt. All resulting reads were reverse complemented and aligned to sequences in the miRNA libraries. Within the duplex, a mismatch contributes 1.0 to the score, unless it is a G-U (wobble) pair, which contributes 0.5 to the score. The predicted targets of the miRNAs from Mireap (https://sourceforge.net/projects/mireap/) were obtained using the rules described by Allen *et al*.^76^ and Schwab *et al*.^77^. The selected targets were categorized as 0, 1, 2, 3 or 4, respectively, asdescribed previously^78^. T-plots were built based on the distribution of signatures (and abundances) along these transcripts.

### iTRAQ analysis

Leaves from the four samples (PF2, PF4, PF2S and PF4S) were ground to powder in liquid nitrogen. Protein extraction, isolation and mass spectroscopy were performed according to the previous methods^79, 80^. The extracted proteins were kept at −80 °C for further analysis.

The samples were labeled with iTRAQ tags as follows (sample, tag): PF2, 113; PF2S, 114; PF4, 115; PF4S, 116; and PF2-2, 117; PF2S-2, 118; PF4-2, 119; PF4S-2,121 for the replicates. Four other samples were labeled with the same tags as repeats. The raw files acquired from Orbitrap were converted into MGF files, which were searched using Mascot against a database containing 29,942 sequences. The search criteria were: full tryptic specificity was required (cleavage after lysine or arginine residues, unless followed by proline); one missed cleavage was allowed; carbamidomethylation (C) iTRAQ8plex (N-term), and iTRAQ8plex (K) were set as fixed modification; Gln- >pyro-Glu (N-term Q), Oxidation (M), and Deamidated (NQ) were applied as variable modifications; and mass tolerance of 0.05 Da (precursor) and 0.1 Da (fragments). The charge states of peptides were set to +2 and +3. The database search results were filtered using peptides with significance scores (≥20) to set the false discovery rate (FDR) to 1% on the peptide and protein levels based on the number of reverse protein sequence hits in the data sets. Each confident protein was identified as involving at least one unique peptide. For protein quantization, a protein had to contain at least two unique peptides. The quality precision of the coupled tandem mass spectrometry of Triple TOF 5600 was controlled at less than 2 ppm. To avoid identification omissions, we set the peptides matching error of the database search strategy under 0.05 Da.

### Analysis of differentially expressed transcripts, the miRNA target genes and different abundance proteins

Expression levels of genes and isoforms were quantified using RNA-Seq by Expectation Maximization to (RSEM)^81^ determine which transcripts were isoforms of the same gene under the benefit of Expectation-Maximization (EM) algorithm^82^. The FPKM method^83^ was used to calculate gene expression by counting fragments.

Based on the Poisson distribution^84^, genes with FDR ≤ 0.001^85^ and |Log2Ratio| ≥ 1 were defined as differentially expressed genes (DEGs).

To identify differences in miRNA expression between two samples, the abundance was analyzed using the strict fold change algorithm^86^ and p*-*values^87^. MiRNAs with |fold changes| ≥ 1, FDR ≤ 0.001 and *P-*values < 0.05 were considered as significantly different expression^88^.

Protein identification and quantification was performed based on the methods of Guo *et al*.^89^. Quantitative protein ratios were weighted and normalized using the median ratio in Mascot. Proteins with p-values < 0.05 and |fold change| ≥ 1.2 was considered as differentially abundant proteins (DAPs).

### Bioinformatics analysis

All the DEGs (including target genes) and DAPs were searched against NCBI’s non-redundant protein sequence database (Nr) using BlastX (http://blast. ncbi.nlm.nih.gov/Blast.cgi) to identify homologous sequences with shared similarity. The best homolog was used to assign gene ontology (GO) annotations (http://www.geneontology.org/) and Clusters of Orthologous Groups (COG) annotations. Similarly, pathway annotations were assigned based on Blastall hits against the Kyoto Encyclopaedia of Genes and Genomes (KEGG) database (http://www.genome.jp/kegg/). Gene numbers for every GO term and Pathway and hyper geometric distribution were used to detect significantly enriched GO terms and pathways with corrected *P*-value ≤ 0.05^90^ as the threshold.

### Quantitative RT-PCR

RNAs were isolated from the PF2 and PF4 plants at four developmental stages under 70 mM NaCl treatment (0, 10, 15 and 20 days acting as S-0, S-1, S-2 and S-3, respectively) using a Plant RNA Extraction Kit (Aidlab Biotechnologies Co., Ltd., Beijing, China), with three biological replications for each stage. The PCR amplification was performed using a SuperScriptIII platinum SYBR Green one-step qRT-PCR kit (Invitrogen, Carlsbad, CA, USA) on a CFX96 real time PCR system (Bio-Rad, Hercules, CA, USA). The PCR conditions were as described by Fan *et al*.^90^, with three technical replicates for each sample. The primers used for the miRNAs and genes are listed in Table 4. The miRNA stem-loop primers were designed as described previously^91^. U6 was the miRNA endogenous reference and Paulownia 18S rRNA was the target gene endogenous reference.

**Table 4:** Primers of quantitative RT-PCR analysis.

GeneID	Sense Primer	Anti-sense Primer
18 s	ACATAGTAAGGATTGACAGA	TAACGGAATTAACCAGACA
PAU024715.1	TTAGATGTAGATGCTCTTC	GATGTGGATATAGGTTGG
PAU013189.1	AGAATATAATGCTGGCTAT	CTCTGGTTATCATCTTCA
PAU025334.3	TGCTGTTGATATTGTTCT	CGATAGATACCTTATTGCTA
PAU000825.1	CCAGGTATAGTGATAATCC	CTTCTTCCATAACATCTCT
PAU009169.1	CCTCATAACCATTCACAT	TTCATTAAGCATCTCATACT
PAU024054.1	AATGTTAGCAATCAGGAA	TAACGCTTATGTCATCAA
PAU027327.1	GTTCTTCCTGTCATTCAA	TTCCTTGAGACCTGTATT
PAU018833.1	TTCCTTCCTACACTTCAG	AATCATCGGTCCATAGTT
PAU013408.2	CCATCAGAATACAAGACA	GCCACAGAACATATAGAA
PAU029564.1	CTGCTATCTACGATTCAA	ATAATGGTGAGGTTGTTG
PAU007545.1	ATTAGTAACTCCTCTGATG	ATACCACTGTCAATAATAGA
PAU018567.1	AGTGATGTTATTATTGTCCT	CTCTCCTTCCTCTATTGA
PAU005576.1	TATAGTTCAAGGTGGACAA	GTTCTTAACAATAGCATCTGA
PAU012903.1	AAGATTGGAAGTGTTAGA	CAGTGATTATTGATGAGTAA
PAU008363.1	TTACAGCAGAACCAGAGAC	AATCCATCAGAGGCAAGG
PAU024067.1	CGAATAACAGATAGCACAAT	CAACATTAGCAACCACAA
PAU002280.1	CGTCGTTATCTTCTCCTT	CCTACTTCCTCATCTTCTC
PAU008956.1	AGTGCTATTACAATCTTGAA	TGTGCTAATTCCTTCCTA
PAU028599.1	GACACATCAGATACTCTAAGG	TAGCATCCATACCACCAT
u6	CTCGCTTCGGCAGCACA	AACGCTTCACGAATTTGCGT
Pfo-miR159a	TTTGGATTGAAGGGAGCTCTA	
Pfo-miR167a	TGAAGCTGCCAGCATGATCTA	
Pfo-miR171b	AGATATTGGTGCGGTTCAATT	
Pfo-miR398a	TGTGTTCTCAGGTCGCCCCTG	
Pfo-M245	AGGTGCAGGTGCTGGTGCAGA	
Pfo-miR160a	GCGTATGAGGAGCCAAGCATA	
Pfo-miR168	TCGCTTGGTGCAGGTCGGGAA	
Pfo-miR172	AGAATCTTGATGATGCTGCAT	

### Data Archiving Statement

The raw sequence data are currently being submitted and the accession numbers will be supplied once available.

## Electronic supplementary material

Supplementary Info Files

Table S1

Table S2

Table S3

Table S4

Table S5

Table S6

Table S7

Table S8

Table S9

Table S10

Table S11

Table S12

Table S13

Table S14

Table S15
