# Supplementary material for: Genome of Paulownia (Paulownia fortunei) illuminates the related transcripts, miRNA and proteins for salt resistance
Source: Sci Rep. 2017 Apr 28;7:1285. doi: 10.1038/s41598-017-01360-9 (PMC5430789; doi:10.1038/s41598-017-01360-9)
Supplement: Supplementary file 1 — Supplementary Info Files [file 41598_2017_1360_MOESM1_ESM.pdf]

Full title:

Genome of Paulownia (*Paulownia fortunei*) illuminates the related transcripts, miRNA and proteins for salt resistance

Authors:

Guoqiang Fan[1,#,*], Limin Wang[1,#], Yanpeng Dong[1,#], Zhenli Zhao[1], Minjie Deng[1], Suyan Niu[1], Xiaoshen Zhang[2], Xibing Cao[1]

Affiliations:

1 Institute of Paulownia, Henan Agricultural University, Zhengzhou, Henan, China

2 Zhengzhou Agriculture & Forestry Scientific Research Institute, Zhengzhou, Henan, China

[#] Equal contributors

[*] Correspondence: Guoqiang Fan, Institute of Paulownia, Henan Agricultural University, Zhengzhou, Henan, China, Tel/Fax: 86+371- 6355-8605,E-mail: zlxx64@126.com.

Supplementary Figures and Tables

Figure S1 Sequencing data assessment. Base composition of clean data in PF2(a1), PF2S(b1), PF4(c1) and PF4S(d1); base quality of clean data in PF2(a2), PF2S(b2), PF4(c2) and PF4S(d2); reads distribution on PF2(a3), PF2S(b3), PF4(c3) and PF4S(d3) genes.

Figure S2 Cluster analysis of DEGs in four comparisons: PF2S *vs* PF2, PF4S *vs* PF4, PF4 *vs* PF2 and PF4S *vs* PF2S.

Figure S3Gene ontology analysis of DEGs in PF2S *vs* PF2(a) and PF4S *vs* PF4 (b).

Figure S4 The detailed information of four samples' proteins. a: Distribution of protein's sequences coverage; b: peptide length distribution; c: protein mass distribution.

Figure S5 COG and Gene ontology analysis (a:COG analysis, b:biological process; c: cellular component; d: molecular function) **of all proteins.**

Figure S6 Gene ontology function classification in all up-regulation(a), all down-regulation(b) and Autotetraploid predominant salt response key proteins(c).

Figure S7 Length distribution of miRNA(a) and the distribution of clean reads (b:PF2, c:PF2S, d:PF4, e:PF4S) **across the different chromosomes.**

Figure S8 The correlation between transcriptome and proteome. Quantitative proteins and genes in PF2S *vs* PF2 (a1), PF4 *vs* PF2 (b1), PF4S *vs* PF2S(c1) and PF4S *vs* PF4 (d1); contrary expressional tendencies between proteins and genes in PF2S *vs* PF2 (a2), PF4 *vs* PF2 (b2), PF4S *vs* PF2S (c2) and PF4S *vs* PF4 (d2); same expressional tendencies between proteins and genes in PF2S *vs* PF2 (a3), PF4 *vs* PF2 (b3), (c3) PF4S *vs* PF2S and PF4S *vs* PF4 (d3).

Table S1 Statistics of each sample comparing with Paulownia genome.

Table S2 Top 20 up and down differentially expressed genes in PF2S *vs* PF2, PF4 *vs* PF2, PF4S *vs* PF4 and PF4S *vs* PF2S.

Table S3 The significantly enriched GO terms of PF2S *vs* PF2 and PF4S *vs* PF4.

Table S4 The KEGG pathway enrichment distribution of PF2S *vs* PF2 and PF4S *vs* PF4.

Table S5 The identified transcription factor families in PF2S *vs* PF2 and PF4S *vs* PF4.

Table S6 Autotetraploid predominant salt response genes.

Table S7 The KEGG pathway enrichment distribution of Autotetraploid predominant salt response genes.

Table S8 The COG function classification and the KEGG pathway classification of all the proteins (a: COG function classification; b:KEGG pathway classification)**.**

Table S9 The consistently different abundance proteins in PF4S *vs* PF2S, PF2S *vs* PF2 and PF4S *vs* PF4.

Table S10 The GO enrichment distribution(a) and KEGG pathway enrichment distribution (b) of Autotetraploid predominant salt response proteins.

Table S11 The miRNA statistics of four samples comparing with Paulownia genome.

Table S12 The identified conserved miRNA and novel miRNA in PF2, PF2S, PF4

and PF4S.

Table S13 The differentially expressed miRNA in PF2, PF2S, PF4 and PF4S.

Table S14 The sequence statistics of degradome comparing with Paulownia genome (a) and the predicted target genes of miRNA(b) .

Table S15 The statistics of miRNA-target mRNA/proteins in PF4S *vs* PF4 and PF2S *vs* PF2.

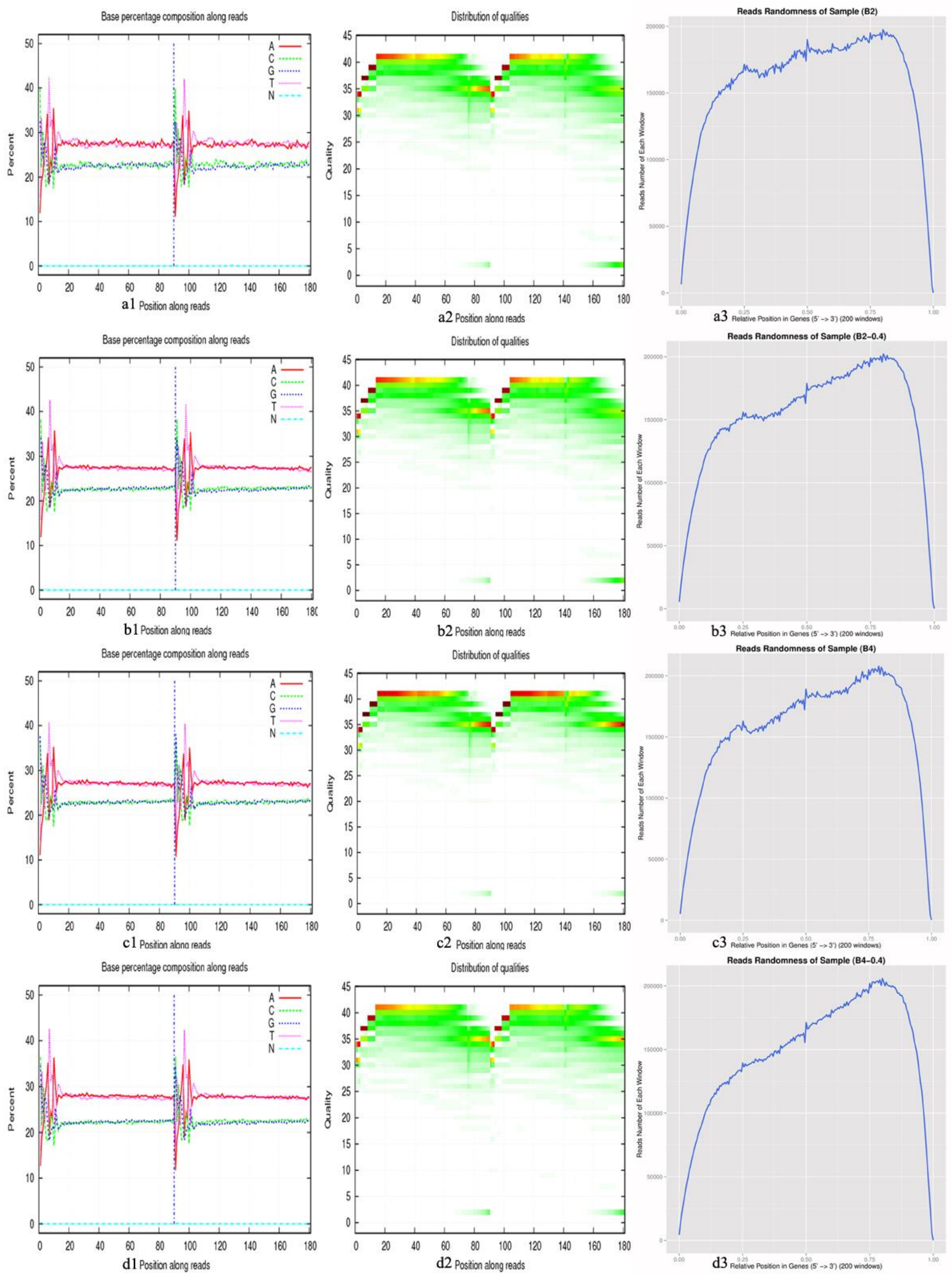

Figure S1 Sequencing data assessment. Base composition of clean data in PF2(a1), PF2S(b1), PF4(c1) and PF4S(d1); base quality of clean data in PF2(a2), PF2S(b2), PF4(c2) and PF4S(d2); reads distribution on PF2(a3), PF2S(b3), PF4(c3) and PF4S(d3) genes.

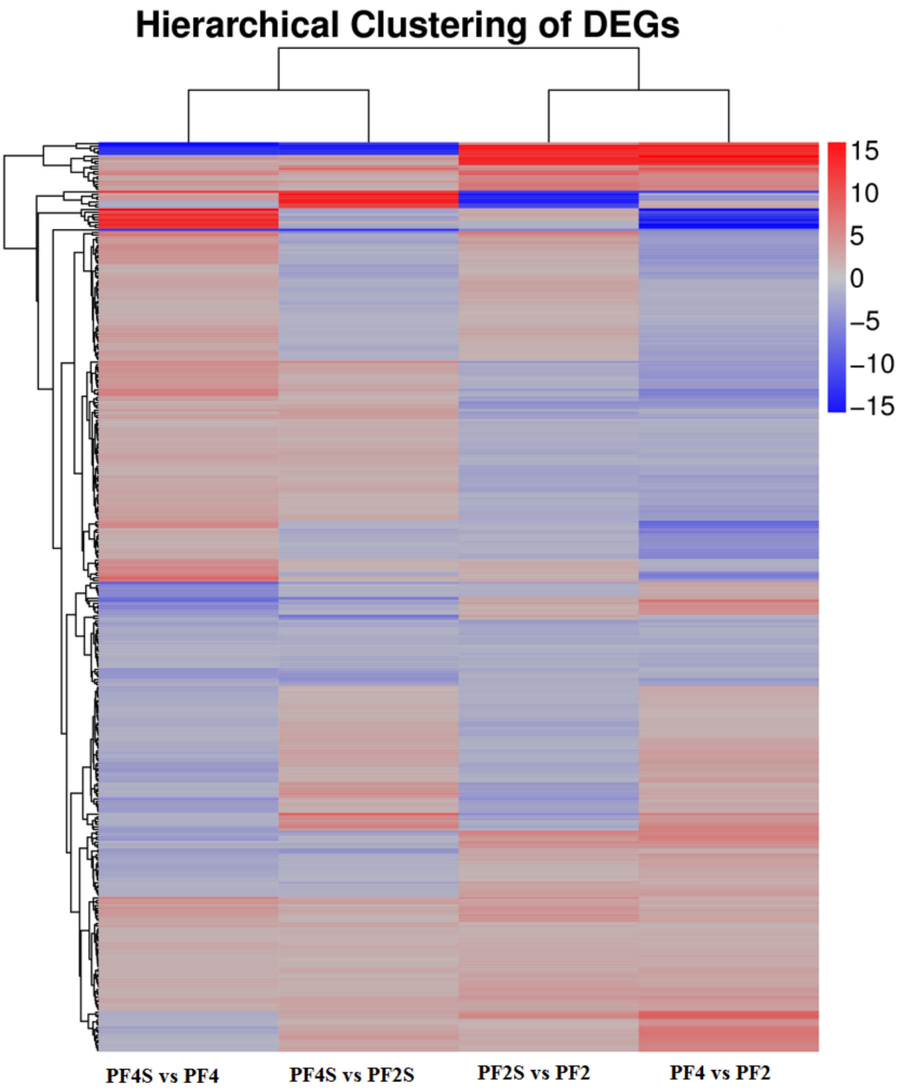

Figure S2 Cluster analysis of DEGs in four comparisons: PF2S *vs* PF2, PF4S *vs* PF4, PF4 *vs* PF2 and PF4S *vs* PF2S.

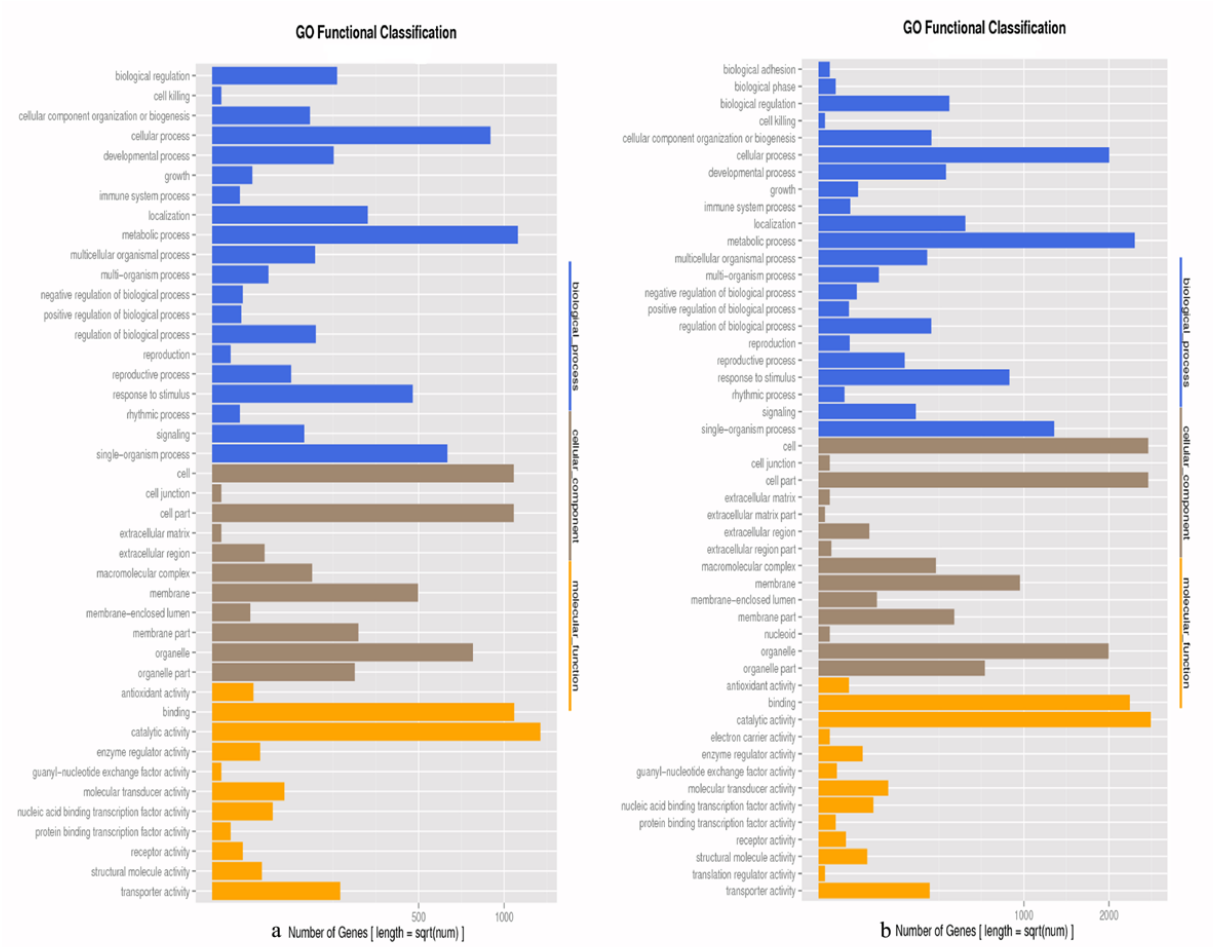

Figure S3 Gene ontology analysis of DEGs in PF2S *vs* PF2(a) and PF4S *vs* PF4 (b).

Figure S4 The detailed information of four samples' proteins. a: Distribution of

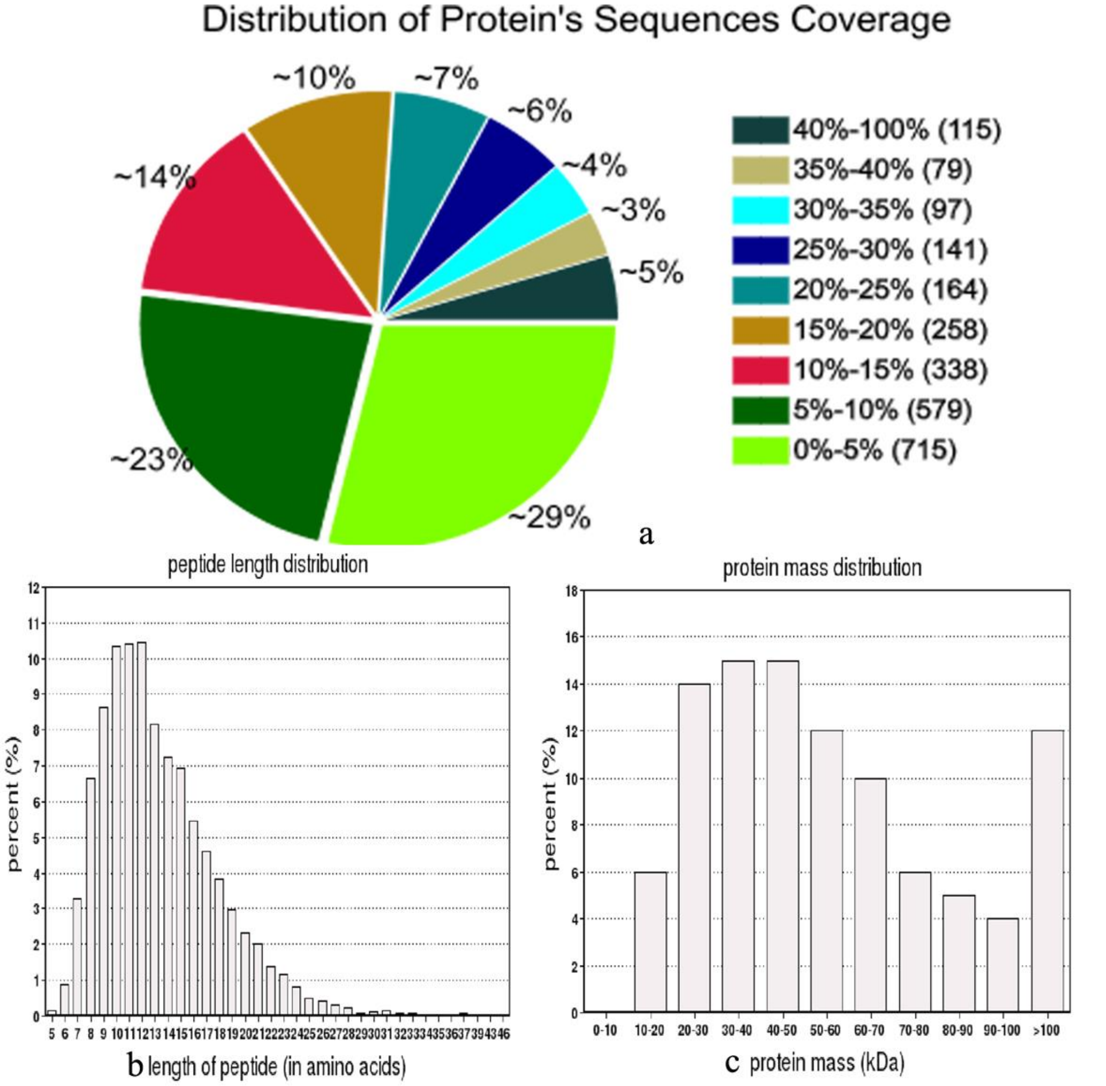

protein's sequences coverage; b: peptide length distribution; c: protein mass distribution.

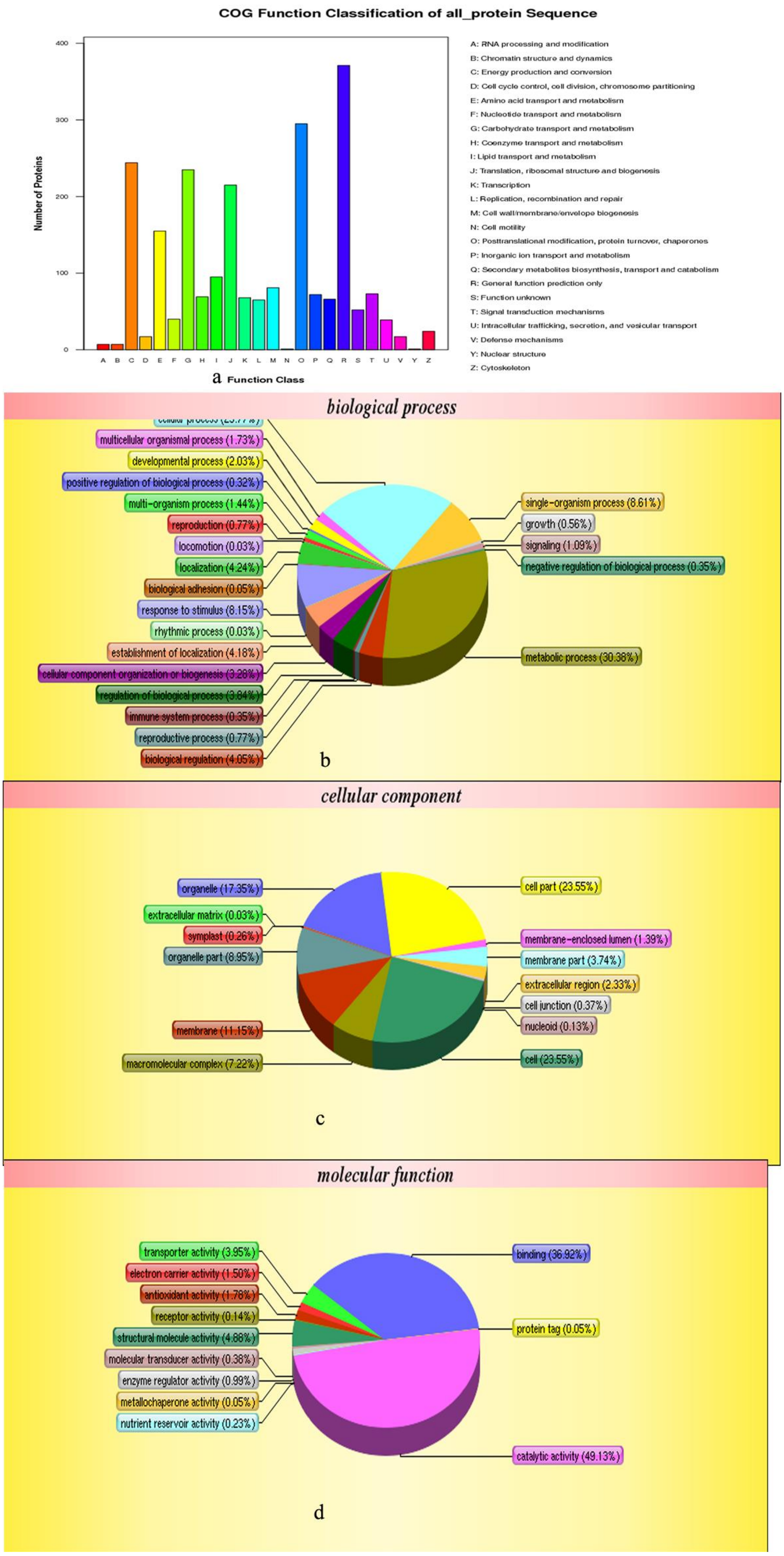

Figure S5 COG and Gene ontology analysis (a:COG analysis, b:biological process; c: cellular component; d: molecular function) **of all proteins.**

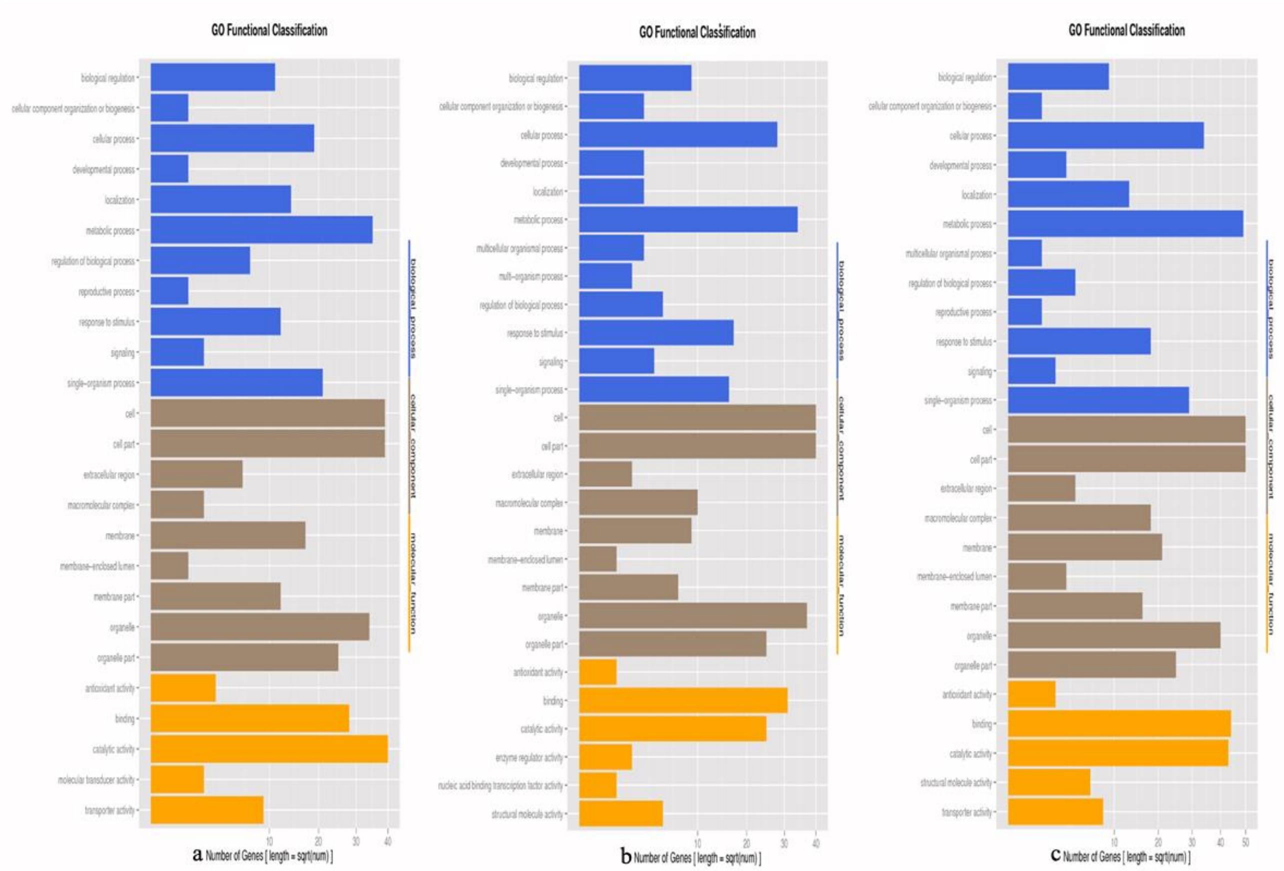

Figure S6 Gene ontology function classification in all up-regulation(a), all down-regulation(b) and Autotetraploid predominant salt response key proteins(c).

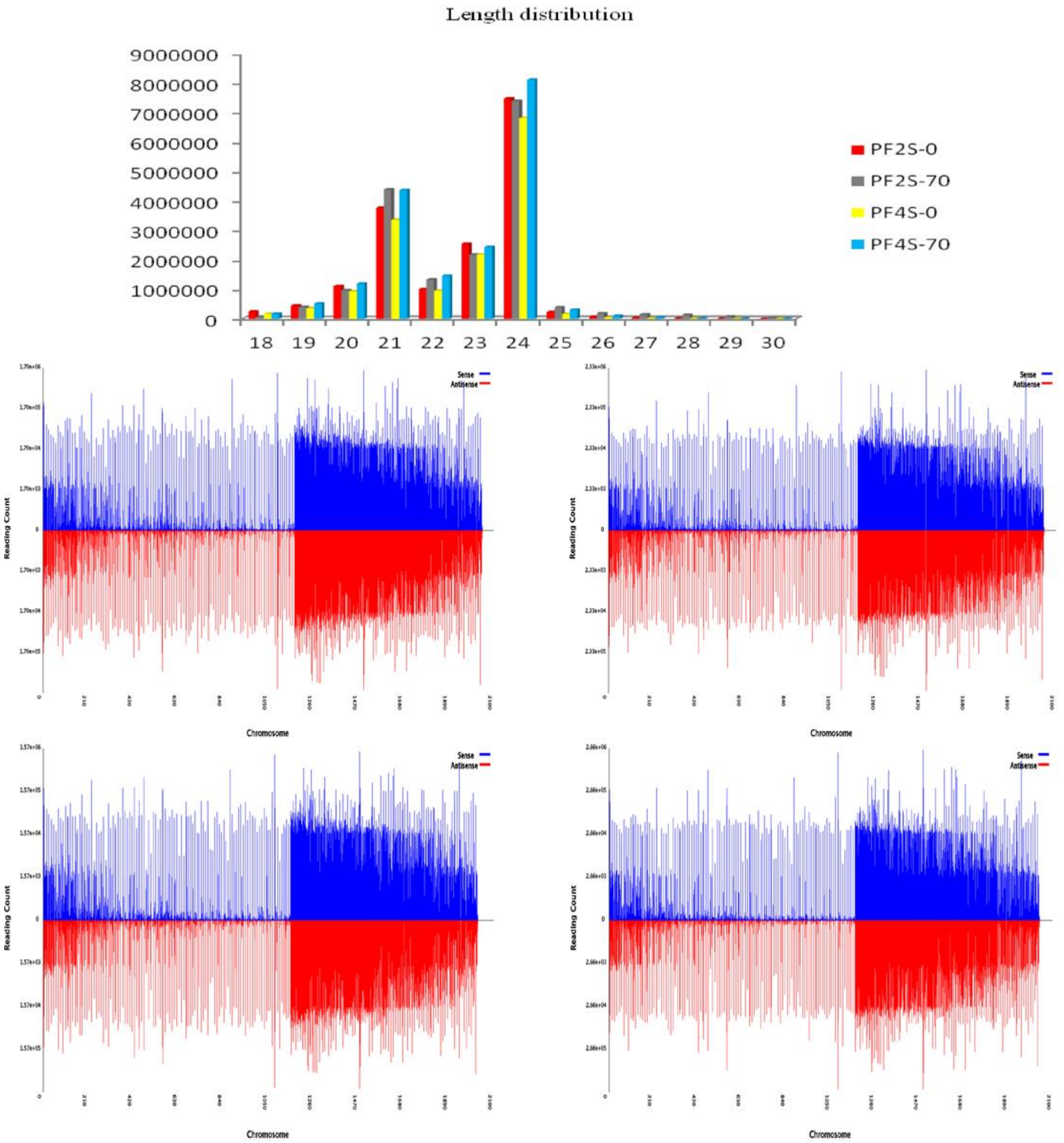

Figure S7 Length distribution of miRNA(a) and the distribution of clean reads (b:PF2, c:PF2S, d:PF4, e:PF4S) **across the different chromosomes.**

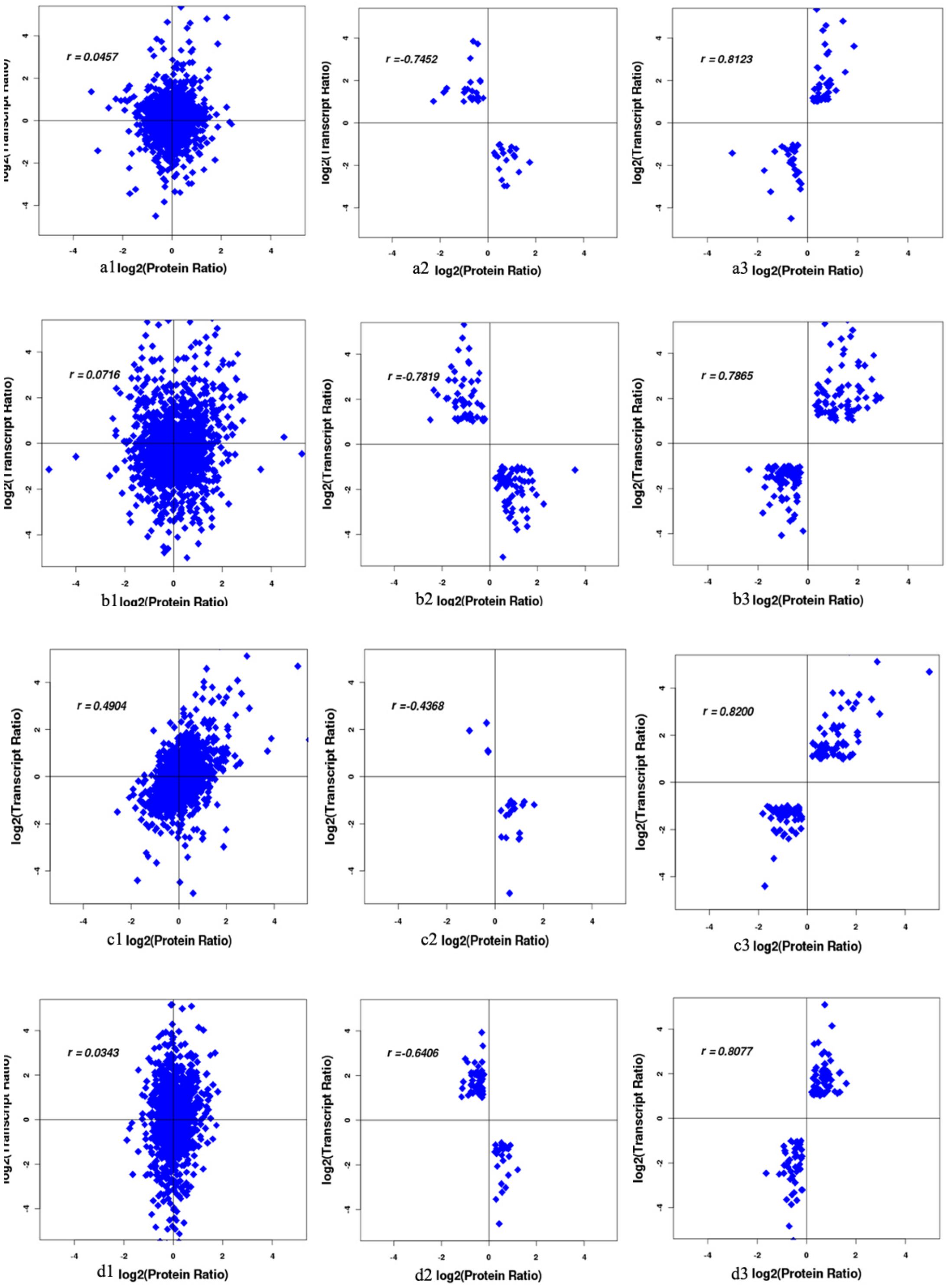

Figure S8 The correlation between transcriptome and proteome. Quantitative proteins and genes in PF2S *vs* PF2 (a1), PF4 *vs* PF2 (b1), PF4S *vs* PF2S(c1) and PF4S *vs* PF4 (d1); contrary expressional tendencies between proteins and genes in PF2S *vs* PF2 (a2), PF4 *vs* PF2 (b2), PF4S *vs* PF2S (c2) and PF4S *vs* PF4 (d2); same expressional tendencies between proteins and genes in PF2S *vs* PF2 (a3), PF4 *vs* PF2 (b3), (c3) PF4S *vs* PF2S and PF4S *vs* PF4 (d3).
